# Prediction of Cu Zeolite NH_3_-SCR Activity from Variable Temperature ^1^H NMR Spectroscopy

**DOI:** 10.3390/molecules28186456

**Published:** 2023-09-06

**Authors:** Sambhu Radhakrishnan, Sam Smet, C. Vinod Chandran, Sreeprasanth Pulinthanathu Sree, Karel Duerinckx, Gina Vanbutsele, Johan A. Martens, Eric Breynaert

**Affiliations:** 1 Centre for Surface Chemistry and Catalysis—Characterization and Application Team (COK-KAT), KU Leuven, Celestijnenlaan 200F Box 2461, 3001 Heverlee, Belgium; 2 NMR/X-ray Platform for Convergence Research (NMRCoRe), KU Leuven, Celestijnenlaan 200F Box 2461, 3001 Heverlee, Belgium

**Keywords:** nuclear magnetic resonance spectroscopy, copper zeolite, SCR catalysis, copper mobility, paramagnetic relaxation enhancement, paramagnetic shift, ^1^H NMR, variable temperature NMR spectroscopy

## Abstract

Selective catalytic reduction (SCR) of NO_x_ by ammonia is one of the dominant pollution abatement technologies for near-zero NO_x_ emission diesel engines. A crucial step in the reduction of NO_x_ to N_2_ with Cu zeolite NH_3_-SCR catalysts is the generation of a multi-electron donating active site, implying the permanent or transient dimerization of Cu ions. Cu atom mobility has been implicated by computational chemistry as a key factor in this process. This report demonstrates how variable temperature ^1^H NMR reveals the Cu induced generation of sharp ^1^H resonances associated with a low concentration of sites on the zeolite. The onset temperature of the appearance of these signals was found to strongly correlate with the NH_3_-SCR activity and was observed for a range of catalysts covering multiple frameworks (CHA, AEI, AFX, ERI, ERI-CHA, ERI-OFF, *BEA), with different Si/Al ratios and different Cu contents. The results point towards universal applicability of variable temperature NMR to predict the activity of a Cu-zeolite SCR catalyst. The unique relationship of a spectroscopic feature with catalytic behavior for zeolites with different structures and chemical compositions is exceptional in heterogeneous catalysis.

## 1. Introduction

Selective catalytic reduction of NO_x_ (SCR) by ammonia is a widespread de-NO_x_ technology for combustion processes [1,2,3]. Cu exchanged zeolites have been demonstrated to be very efficient NH_3_-SCR catalysts above 473 K, and retain a reasonable activity at lower temperatures, explaining their widespread use for mobile applications such as diesel engines [2,4,5]. Prime examples are Cu exchanged small-pore zeolites with the chabazite (CHA) framework topology. They successfully mediate the complex multi-electron, multi-proton redox reactions, contributing to the overall NH_3_-SCR reaction: 4NO + 4NH_3_ + O_2_ → 4N_2_ + 6H_2_O [6,7,8,9,10]. Suggested reaction pathways involve the oxidation of NO to NO_2,_ activating NO_x_ for faster NH_3_-SCR [11,12]. Given the large number of electrons and atoms involved in the catalytic cycle, it is unlikely that a single Cu-atom can function as the active site [2]. Based on reaction kinetics and DFT calculations, binuclear cationic species such as [Cu^I^(NH_3_)_2_]^+^-O_2_-[Cu^I^(NH_3_)_2_]^+^ have been proposed as reaction intermediates [4,13,14,15,16]. Transient XAS measurements revealed a second order dependence on Cu density for the O_2_-assisted oxidation of [Cu^I^(NH_3_)_2_]^+^. Such co-operative effects are consistent with the formation of paired Cu sites [5,16,17]. Evidence for dimer formation has also been obtained using DFT assisted fiber-optic UV-vis-NIR spectroscopy [18,19] and XAS spectroscopy [13]. Recently, Wenshuo et al. revealed the importance of Cu^II^ pair formation in the reduction half cycle by NH_3_-TPD and DFT calculations [20]. In the proposed mechanism, NO oxidative activation to mobile nitrite-intermediates accounts for the reduction of Cu^II^ to Cu^I^. The rate of the reaction was shown to quadratically depend on the Cu^II^ concentration, confirming the importance of Cu pairing to generate the active site.

Recent approaches to identify the Cu-speciation in Cu-CHA zeolites involves a combination of *in situ* Electron Paramagnetic Resonance spectroscopy and H_2_ Temperature Programmed Reduction studies [21,22]. In hydrated zeolites and avoiding Cu-loadings close to the cation exchange capacity, all Cu-species (Z_2_Cu^II^, ZCu^II^OH where Z represents AlO_4_^-^ units in the zeolite) can be observed with EPR. Only in the case of very high Cu-loadings, small amounts of EPR silent CuO or other types of clustered species were detected. The low concentration of paired Cu ions in synthesized catalysts implicates Cu ion mobility as a key factor in generating the active site. Based on ab-initio molecular dynamics calculations, Göltl et al. concluded that thermal motion of Cu^2+^ already occurs at temperatures as low as 300 K [23,24]. The predicted Cu-ion mobility and change of coordination site also explains the observation of complex FTIR spectra for a single NO molecule adsorbed to a single active site [24,25]. Asides being linked to Cu pairing, the SCR catalytic activity has also been shown to correlate to the fraction of [Cu(OH)]^+^ exchanged onto the zeolite [26]. Because of its weaker coordination to the framework, as compared to Cu^2+^, this species is potentially mobile and could assist transient formation of Cu pairs. Using H_2_ TPR [Cu^II^OH]^+^ species were also shown to more easily reduce as compared to exchanged Cu^2+^, suggesting a higher redox activity of the [Cu^II^OH]^+^. In a dehydrated state, only a fraction of the [Cu^II^OH]^+^ species observed in the hydrated state remains EPR visible. Disappearance of part of the signal has been attributed to the pseudo Jahn–Teller effect [27]. Even though the methodology combining EPR in hydrated and dehydrated states with H_2_ TPR could identify changes in the Cu-speciation, no correlation between speciation and catalytic activity of the respective catalysts was reported. Under low-temperature SCR reaction conditions, NH_3_-coordination has also been hypothesized to affect catalytic performance by enabling transient pairing of Cu ions residing in adjacent zeolite cages [16,28,29]. Partial hydration (outer-sphere exchange) or ligation with NH_3_ and/or NO has indeed been documented to significantly weaken the coordination of Cu ions to zeolite framework oxygens, thus enhancing Cu mobility [30,31,32,33,34].

While computational chemistry suggests a strong correlation between Cu ion mobility and NH_3_-SCR catalytic activity, experimental evidence has been limited to a small number of samples [5,13,28,35]. A practical method to experimentally measure the mobility of Cu in Cu exchanged zeolites could therefore assist in experimentally confirming the dominant role of Cu-ion mobility in NH_3_-SCR catalysis. Based on a series of spectroscopic measurements and catalytic tests on a range of zeolite samples covering multiple framework types (CHA, AEI, AFX, ERI, ERI-CHA, ERI-OFF, *BEA) and copper contents (Cu/Al ratios of 0.11 to 0.36), this report proposes variable temperature ^1^H NMR spectroscopy as a suitable tool to experimentally measure the mobility of Cu in zeolites. The results even suggest its potential as a predictive tool for catalytic activity.

## 2. Results

**Figure 1:**
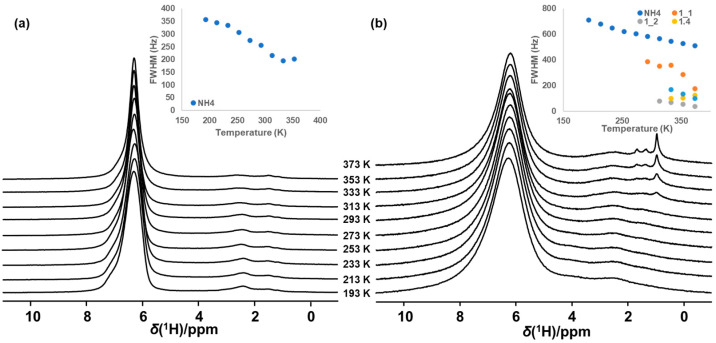
Variable temperature (VT) ^1^H NMR spectra of (**a**) NH_4_-exchanged CHA-1, Inset: FWHM of the NH_4_-resonance as function of temperature and (**b**) Cu-exchanged CHA-1 (CHA-1H Cu/Al 0.3). Inset: FWHM of the NH_4_-resonance and suddenly appearing resonances at ~1 ppm (which can be decomposed into 2 components, labeled as 1_1 and 1_2), 1.4 ppm and 1.7 ppm, respectively).

Zeolite based NH_3_-SCR catalysts are typically only partially ion exchanged with Cu^II^ ions. The other exchange sites remain charge compensated by Brønsted acid protons or by protonated species such as chemisorbed NH_3_ [2,6,14]. Figure 1 shows the temperature dependence (193–373 K) of the quantitative direct excitation ^1^H MAS NMR spectra of the NH_4_- and Cu-exchanged forms of a representative, vacuum dried (1 mbar, 200 °C, 16 h) chabazite zeolite that can be turned into a performant NH_3_-SCR catalyst (sample CHA-1H). Both in the NH_4_^+^ zeolite and in the partially Cu exchanged catalyst, chemisorbed NH_3_ (or ion exchanged NH_4_^+^) is clearly visible at 6.4 ppm. Comparing both spectroscopic series, the broadening effect also resulting from the presence of paramagnetic Cu^II^ in the catalyst sample is readily observed. Each series of ^1^H MAS NMR spectra also allows for the *in situ* probe of the temperature dependent proton dynamics.

In the series for the NH_4_ exchanged zeolite (Figure 1a), broadening (and narrowing) of resonances as a function of temperature is predominantly associated with two effects: mobility and chemical exchange. Figure 1a reveals the impact of both of these effects. As temperature increases from 193 to 313K, the ^1^H NMR signal associated with the chemisorbed NH_3_ exhibits the impact of motional narrowing as revealed by the evolution of the full width at half maximum (FWHM) of the resonance (Figure 1a inset). Inversely, as the ^1^H mobility increases with increasing temperature, the ^1^H resonance at 2.5 ppm, associated with aluminols (AlOH), broadens as a result of enhanced chemical exchange, a process readily revealed by ^1^H EXchange SpectroscopY (EXSY) (Figure S1). The EXSY spectrum shown in Figure S1 indeed clearly shows the off-diagonal exchange correlations.

In addition to the temperature effects also observed for the NH_4_-form of the zeolite (Figure 1a), comparison of the spectroscopic series in Figure 1a,b readily reveals the appearance around 300 K of sharp ^1^H NMR resonances with a chemical shift between 1 and 2 ppm, exclusively in the Cu-exchanged catalyst. The sudden appearance of these resonances is fully reversible and is never observed in the purely NH_4_-exchanged material. In addition to the already described broadening (and narrowing) effects on the ^1^H NMR spectra shown in Figure 1a, in presence of paramagnetic ions (e.g., Cu^II^), two additional effects, paramagnetic relaxation enhancement (PRE) and paramagnetic shift (PS), can impact spectral broadening and shifting of NMR resonances [36]. The extent of the paramagnetic influence varies largely on the proximity of the paramagnetic center to the observed nucleus and on its residence time in the vicinity of the nuclear spin. The PRE effect arises from the hyperfine interactions between the unpaired electrons in the paramagnetic center and a NMR active nucleus in its vicinity, resulting in enhanced relaxation [37]. Depending on the distance to the NMR nucleus (proportional to γ^2^/r_0_^6^) and mobility of the paramagnetic center, enhanced *T*_2_ relaxation is observed as broadening and ultimately as blinding (disappearance) of resonances in the 1D spectra [36,37,38]. Comparison of Figure 1a,b readily demonstrates how the presence of Cu^II^ ions causes broadening of the NH_4_ resonance at 6.4 ppm, an effect that increases with an increasing concentration of paramagnetic Cu^II^ in the sample (Figure S2). As the Cu loading is increased from 0 to 2.5 and 3.2 wt.%, corresponding to a Cu/Al ratio of 0.23 and 0.30, respectively, FWHM of the NH_4_ resonance increases from ca. 250 Hz in the absence of Cu ions to, respectively, ca. 545 Hz and 633 Hz in the Cu containing samples. Besides giving rise to broadening effects, paramagnetic electron spins can also affect local magnetic fields felt by NMR active nuclei of interest [38,39], shifting their resonance frequency. This phenomenon is called paramagnetic shift scales with γ^2^/r_0_^3^. It is readily observed in the ^1^H NMR spectrum of a Cu loaded zeolite after exposure to NH_3_ gas. Figure S3 displays a broad signal in the negative ppm range that increases with increasing temperature while simultaneously the resonance of chemisorbed ammonium (6.4 ppm) decreases in intensity. The broad signal at negative chemical shifts has previously been reported to appear upon NH_3_ adsorption on Cu exchanged zeolite Y [40], and has been identified by ^1^H-{^63^Cu} TRAnsfer of Population in DOuble-Resonance (TRAPDOR) [41] NMR (Figure S4) as NH_3_ coordinated to Cu^II^ which is in chemical exchange with ammonia chemisorbed on the Brønsted acid sites. Close inspection of the chemical shift of this species reveals a clear dependence on temperature (Figure S3), a feature typically expected for a resonance impacted by the paramagnetic shift. The signal of NH_3_ ligated to Cu^II^ consequently displays both PRE and paramagnetic shift effects.

**Figure 2:**
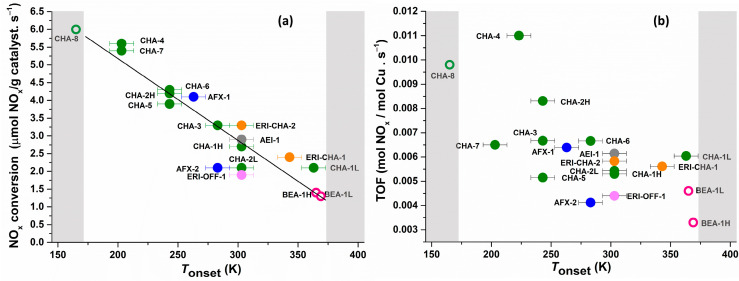
Correlation between the (**a**) NO_x_ conversion and (**b**) turn over frequency (TOF) of Cu-exchanged chabazite catalysts in low temperature (448 K) Cu-zeolite NH_3_-SCR catalysis and the onset temperature (*T*_onset_) for the appearance of new sharp ^1^H NMR signals in quantitative VT ^1^H MAS NMR. The operating temperature range of the MAS NMR probehead is highlighted in white. The estimated values based on a linear fit of the Activity Vs *T*_onset_ plot (y = 9.782 − 0.023*x; R^2^ = 0.87) is indicated in open symbols.

The sudden, temperature induced, reversible appearance of sharp new resonances in the ^1^H direct excitation spectra of Cu-exchanged SCR catalysts (Figure 1b) are clearly related to the presence of Cu on the catalyst. These resonances are never observed for the purely NH_4_ exchanged zeolite and they are narrower than any other ^1^H resonance in the spectrum. In the example of Figure 1b, the appearance of new resonances occurs between 293 K and 313 K. The median of the temperature interval where the new resonances appear is from here on defined as *T*_onset_. In the case of the example in Figure 1b, *T*_onset_ is 303 ± 10 K. A similar sudden appearance of sharp resonances was observed for a wide range of SCR catalysts, covering multiple zeolite frameworks, a range of Si/Al ratios and a range of Cu loadings (Appendix A). Correlating *T*_onset_ for each catalyst with its low temperature SCR catalytic activity reveals an interesting trend (Figure 2). As shown in Figure 2a, *T*_onset_ and NO_x_ conversion clearly exhibit a strong, inverse correlation across all samples evaluated: the lower *T*_onset_, the higher the low temperature NH_3_-SCR NO_x_ conversion of the catalyst. ^27^Al MAS NMR spectra were recorded for all samples and indicated the concentration of extraframework Al was not only negligible, but there also was no correlation between this concentration and the observed *T*_onset_ or the catalytic performance.

Unexpectedly, the correlation between *T*_onset_ and the overall catalytic activity of the sample obfuscates when the activity is expressed as the Cu turn over frequency (TOF) (Figure 2b). This readily reveals that while *T*_onset_ clearly can be used as a proxy for the overall catalytic performance of the catalyst, only a limited fraction of the Cu atoms in the sample is contributing to the catalytic conversion and in extension to the sudden appearance of the sharp ^1^H MAS NMR resonances.

Loading a single zeolite sample (e.g., CHA-1) with low (L) and high (H) amounts of Cu, yielding Cu/Al ratios of 0.21 and 0.30, respectively, impacts both *T*_onset_ and NO_x_ conversion at 448 K. The sample with the highest Cu concentration (CHA-1H) showed the lowest *T*_onset_ (303 ± 10 K) and the highest NO_x_ conversion (2.7 µmol NO_x_/g catalyst. s^−1^) (Figure 1b), while the less active CHA-1L (Figure S6) sample exhibited a *T*_onset_ of 343 ± 10 K and a NO_x_ conversion of 2.1 µmol NO_x_/g catalyst. s^−1^. A similar observation was made for CHA-2, with CHA-2H exhibiting a NO_x_ conversion of 4.2 µmol NO_x_/g catalyst. s^−1^ and a *T*_onset_ of 243 ± 10 K (Figure S5) while CHA-2L showed a NO_x_ conversion of 2.1 µmol NO_x_/g catalyst. s^−1^ and a *T*_onset_ of 303 ± 10 K. Increasing the Cu concentration within a single zeolite increases the catalytic activity while decreasing *T*_onset_.

For some catalyst samples, the peak narrowing transition occurred outside of the experimentally accessible temperature window of the NMR probe head (173–373 K). In the case of CHA-8, the most active catalyst in this study (6 µmol NO_x_/g catalyst. s^−1^), a resonance at ca. 0 ppm undergoes narrowing with increasing temperature (Figure S7). As this resonance is however already present at the lowest temperature accessible by the NMR probe head (173 K), determination of *T*_onset_ is difficult. For the samples with lowest catalytic activity BEA-1L, BEA-1H (1.2 and 1.3 µmol NO_x_/g catalyst. s^−1^, respectively), *T*_onset_ could not be determined experimentally as no narrowing was observed in the temperature range of the probe-head. Based on a linear fit of the correlation between NO_x_ conversion and *T*_onset_ data presented in Figure 2a, expected values for *T*_onset_ for samples CHA-8, BEA-1L, BEA-1H were estimated (Figure 2b). For all these samples, the estimation of *T*_onset_ using their respective NO_x_ conversions, indeed falls too close to or outside of the temperature window accessible by the NMR probe head to enable experimental determination.

**Figure 3:**
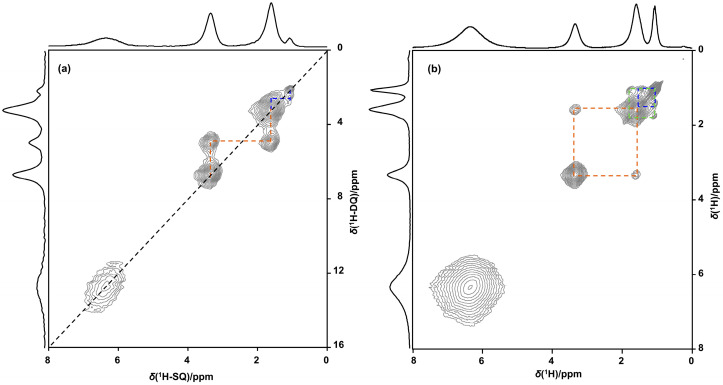
(**a**) ^1^H-^1^H DQ-SQ and (**b**) ^1^H-^1^H EXSY NMR spectrum of Cu/NH_4_-CHA-2H at 353 K.

^1^H-^1^H double quantum-single quantum (DQ-SQ) correlation spectroscopy (Figure 3) revealed that the new ^1^H resonances are associated with a chemical environment containing at least two identical protons residing very closely together in space. DQSQ further reveals that all of the new resonances in addition to a DQ self-correlation also exhibit DQ cross-correlations to each other. This implies they are all part of the same local chemical environment. Based on the quantitative direct excitation ^1^H NMR spectra, the concentration of these local chemical environments can be estimated. For the example shown in Figure 1b, at 353 K there are 0.05 mmoles/g of such sites generated as compared to 1.65 meq/g ion exchange sites. In this sample, Cu^2+^ nominally charged compensates 60% of the CEC, while the remaining 40% is compensated by NH_4_^+^. It is clear that these resonances are associated with the reversible generation of a new chemical environment, in a very low concentration both as compared to the concentration of cation exchange sites and to the overall concentration of Cu on the catalyst. This readily explains why the correlation between *T*_onset_ and overall catalyst activity obfuscates when the activity is expressed as a Cu TOF.

Evaluating the observations, only few mechanisms could potentially explain the sudden appearance of sharp resonances:

***Cu^II^ ligation:*** The appearing signals could originate from a temperature induced ligand exchange process. Molecules originally adsorbed onto the zeolite could coordinate with Cu^II^ and exhibit a chemical shift impacted by a paramagnetic shift effect. Since the Cu-zeolite was evacuated at 473 K prior to the measurement, the only possible ligands would be chemisorbed ammonia (*δ*(^1^H) 6–8 ppm) [42], strongly adsorbed water (*δ*(^1^H) 4–6 ppm) [43,44] or the zeolite framework protons, i.e., Brønsted acid site protons (*δ*(^1^H) 3.6–7 ppm) or defect protons (*δ*(^1^H) 0–3 ppm) [43,44]. The new resonances are however sharper than most other resonances in the spectra, indicating they are not broadened by PRE effects. The chemical shift of the new resonances also appears to be independent of temperature. The combination of both observations renders it very unlikely that the signals are derived from a ligand of a paramagnetic Cu^II^ ion.

***Cu^II^ reduction:*** If a Cu^II^ ion suddenly reduces to Cu^I^, proton resonances previously blinded by paramagnetically enhanced *T*_1_ and/or *T*_2_ relaxation can suddenly become visible as the paramagnetic effect disappears. The impacted proton spins should be present in close vicinity of this Cu^II^ atom, either on the exchanger or on a Cu ligand to exhibit such effects. This also implies the new signals should be affected by a ^1^H-{^63^Cu} TRAPDOR NMR experiment, which specifically exploits the vicinity of the quadrupolar Cu atom to induce enhanced relaxation of nearby ^1^H spins, thus impacting the area of the resonances associated with these spins. As shown in Figure S4 this is not the case, readily excluding this option.

***Cu^II^ dimerization:*** When two monomeric Cu species form a dimer exhibiting antiferromagnetic or weak ferromagnetic coupling between the Cu^II^ unpaired electron spins, previously blinded ^1^H resonances could suddenly appear because the paramagnetic effects of the Cu atoms diminish or completely vanish. As for the case of Cu^II^ reduction (supra), this would also imply the new resonance should react to a ^1^H-{^63^Cu} TRAPDOR NMR experiment. As this is not the case (Figure S4), this option can also be excluded.

***Framework defects:*** An alternative explanation, considering the chemical shift of the new resonances, is that these signals originate from framework defects, either reversibly generated or reversibly forming a surface complex with Cu^II^. In the former option, the presence of Cu induces strain in the zeolite framework, catalyzing reversible hydrolysis of siloxane bonds with temperature. In this case, the hydrolysis either occurs at suitable distance for Cu^2+^ not to blind the resonances associated with these defects or Cu^2+^ should exhibit a high enough mobility in the pore space to average out its paramagnetic relaxation enhancement effects. In the latter case, defects generated during the synthesis of the catalyst are blinded at low temperature due to PRE effects resulting from their association with Cu^2+^. Raising temperature, enhanced Cu^2+^ mobility would then again average out its PRE effects, causing the re-appearance of the respective resonances. In zeolites, silanol or aluminol groups associated with framework defects always occur in proton nests. This would explain the double quantum (DQ) correlations observed for the new signals. The previously calculated very low concentration of these sites, as compared to the exchange site concentration and the Cu loading, also suggests that if the generation of such defects would impact the Cu speciation, its impact would be very limited. The sharpness of the signals, in combination with the absence of a ^1^H-{^63^Cu} TRAPDOR (Figure S4) response suggests that even though the generation of the defects is clearly dependent on the presence of Cu, once generated, the Cu atoms either reside at a distance far enough from the defect to minimize their paramagnetic influence or their mobility is high enough to, on average, diminish or cancel out their PRE effects. The occurrence of Cu mobility at such low temperatures might appear surprising, but is nevertheless in full agreement with the theoretically predicted mobility of Cu^2+^ ions in zeolites, even in absence of ligating molecules [23].

## 3. Conclusions

The generation of a multi-electron donating active site is a crucial step in the selective catalytic reduction of NO_x_ by ammonia. In the case of Cu zeolites as NH_3_-SCR catalysts, the required permanent or transient pairing of Cu atoms has been implied to rely on Cu atom mobility. This report demonstrates how variable temperature ^1^H NMR spectroscopy reveals the temperature dependent appearance of sharp ^1^H resonances on Cu zeolite SCR catalysts associated with a very low concentration of sites as compared to the exchange capacity of the zeolite and to its Cu loading. The onset temperature of this effect was found to strongly correlate with the catalytic activity of the investigated samples expressed as conversion of NO_x_ molecules per gram. This correlation was found for a range of catalysts based on multiple zeolite frameworks (CHA, AEI, AFX, ERI, ERI-CHA, ERI-OFF, *BEA), with different Si/Al ratios and different Cu contents, but surprisingly the correlation obfuscates when the catalytic activity is expressed as a Cu turnover frequency. The results point towards universal applicability of variable temperature NMR to predict the activity of a Cu-zeolite SCR catalyst. The unique relationship of a spectroscopic feature with catalytic behavior for zeolites with even different structures and chemical compositions is exceptional in heterogeneous catalysis.

## 4. Materials and Methods

Zeolite samples from different framework types, obtained by using small scale synthesis (CHA, AEI, AFX, ERI, ERI-CHA, ERI-OFF) or purchased (*BEA from Zeolyst), were converted into NH_3_-SCR catalysts by subsequent calcination, ammonium ion exchange and Cu^II^ ion exchange. Details on the synthesis conditions and properties of these zeolite catalyst are provided in Table A1. The crystallinity and phase-purity of all samples was verified with XRD and SEM (Supplementary information Section S2). Following an acid destruction with aqua regia and hydrofluoric acid, elemental analysis was performed using ICP-OES. The low temperature NH_3_-SCR catalytic activity of the Cu catalysts expressed as NO_x_ conversion and turn-over frequency at 175 °C, was evaluated using a synthetic gas mixture comprising 1000 ppm NO, 900 ppm NH_3_, 5 vol% O_2_, 2 vol% CO_2_, 2.2 vol% H_2_O and N_2_ in a fixed bed laboratory reactor at 450 K and gas hourly space velocity (GHSV) of 340,000 h^−1^ (Table A1 and Supplementary Section S1) [45,46]. A wide range of activities (between 1.3–6.0 µmol of NO_x_ per gram of material per second) was observed for this score of catalysts covering a range of zeolite framework types and copper contents (CHA-1H&L and CHA-2H&L).

**Low temperature catalytic activity measurements (NO_x_ conversion)**: An amount of 20 mg of zeolite pellets was diluted with 80 mg broken quartz particles of similar size range and loaded as a fixed bed in a quartz reactor tube with an internal diameter of 4 mm. The bed of catalyst pellets was held in place by quartz wool plugs. The reactor tube was introduced in a tubular furnace. Gas in- and outlets were heated to avoid condensation. First, the catalyst was heated to 450 °C at 5 °C min^−1^ and kept isothermal for 2 h under a gas flow composed of 5% O_2_ and 2.2 vol% H_2_O in N_2_ at a flow rate of 250 mL min^−1^. Subsequently, the catalyst was cooled to 175 °C. An exhaust gas mimic composed of 1000 ppm NO, 900 ppm NH_3_, 5 vol% O_2_, 2 vol% CO_2_, 2.2 vol% H_2_O and N_2_ serving as carrier gas was used for the kinetic evaluation. The gas hourly space velocity was 340,000 h^−1^. The reactor outlet gas composition was analyzed online with an ABB Limas 11HW UV analyzer for NO, NO_2_, SO_2_, and NH_3_, and an ABB Uras 26 NDIR analyzer for N_2_O, CO_2_ and CO.

**Error determination and reproducibility of activity measurement**: In order to verify the reproducibility of the activity measurements, the catalytic activity of CHA-6 was measured multiple times in the reactor and reported below (Table 1). Each time, a loading of a new batch of catalysts was used.

**NMR Investigations:** Direct excitation ^1^H NMR spectra were acquired on a Bruker Avance III 500 MHz (11.7 T) spectrometer equipped with a 4mm H/X/Y triple resonance solid-state magic angle spinning (MAS) probe. Catalyst samples were packed into a 4 mm ZrO_2_ rotor and dehydrated in the rotor for 16 h at 200 °C under vacuum (1 mbar). The rotors with the dehydrated catalysts were capped with vespel snap-on caps and spun at 15 kHz. ^1^H direct excitation spectra were recorded in a quantitative way with a π/2 radio-frequency pulse (RF) at 83 kHz, averaging 8 transients with a recycle delay of 10 s. The samples were equilibrated at the measurement temperature for 20 min. The spectra were referenced to adamantane ^1^H resonance at 1.81 ppm. ^1^H–{^63^Cu} TRAnsfer of Population in DOuble-Resonance (TRAPDOR) nuclear magnetic resonance (NMR) was used to study copper-proton proximity [41]. The experiment was conducted in comparison with a standard Hahn-echo experiment [47]. Both experiments were conducted under MAS conditions with a speed of 15 kHz with an echo delay of 10 µs. For the ^1^H echo, a 2.95 μs 90° pulse and a 5.9 μs 180° pulse was used. The effect of ^63^Cu continuous wave (CW) irradiation on the echo intensity was compared to a full ^1^H echo without ^63^Cu irradiation over the echo duration. ^1^H–^1^H double-quantum–single-quantum (DQ–SQ) MAS correlation spectra were measured using the BABA [42,48] sequence with excitation and conversion periods of 0.13 ms. The two-dimensional spectra were collected with 200 *t*_e_ increments of 66.67 μs in the indirect dimension and 16 transients in the direct dimension. ^1^H–^1^H 2D exchange spectroscopy (EXSY) [49] was performed with a mixing time of 40 ms. The two-dimensional spectra were collected with 400 *t*_e_ increments of 100 μs and 16 transients in the direct dimension. ^1^H decoupled ^27^Al NMR spectra were recorded with a 15° radio frequency pulse of 125 kHz, relaxation delay of 1s, 1024 transients and a SPINAL64 [50] ^1^H decoupling of 56 kHz.

## Figures and Tables

**Table 1:** Catalytic activity data of CHA-6.

Exp.	NO_x_ Conversion (µmol NO_x_ g Catalyst^−1^ s^−1^)
1	4.2
2	4.6
3	4.4
4	4.4
5	4.4
6	4.4
7	4.3

## Data Availability

All the raw NMR data will be made available via Harvard Dataverse.

## Supplementary Materials

The following are available online at https://www.mdpi.com/article/10.3390/molecules28186456/s1: Extra NMR analysis data (VT ^1^H NMR data of CHA-1, CHA-1L, CHA-2, CHA-8, NH_3_-exposed CHA-^1^H, ^1^H-{^63^Cu} TRAPDOR), Zeolite characterization (XRD, SEM) data is provided.

## Appendix A

**Table A1:** Details of NH_3_-SCR catalysts, NH_3_-SCR activity and onset temperature of ^1^H NMR spectral narrowing (*T*_onset_). All the catalysts were partially Cu-exchanged from NH_4_-form.

Sample	Synthesis Details		Si/Al Ratio	Cu/Al Ratio	NO_x_ Conversion (µmol NO_x_/g Catalyst. s)	TOF (mol NO/mol Cu. s^−1^)	*T*_onset_ (K)
	Refs.	Comments					
CHA-1H	[51]		9.1	0.3	2.7	5.3 × 10^−3^	303 ± 10
CHA-1L	[51]		9.1	0.21	2.1	6.0 × 10^−3^	343 ± 10
CHA-2H	[51]		8.8	0.3	4.2	8.3 × 10^−3^	243 ± 10
CHA-2L	[51]		8.8	0.23	2.1	5.4 × 10^−3^	303 ± 10
CHA-3	[52]		6.4	0.22	3.3	6.7 × 10^−3^	283 ± 10
CHA-4	[51,53]	Mixed gel synthesis	4.8	0.17	5.6	1.1 × 10^−2^	223 ± 10
CHA-5	[51]		7	0.36	3.9	5.2 × 10^−3^	243 ± 10
CHA-6	[53]		4.8	0.22	4.3	6.7 × 10^−3^	243 ± 10
CHA-7	[54]	Based on sample Cu-SSZ-13	5.5	0.32	5.4	6.5 ×10^−3^	203 ± 10
CHA-8	[53]	Synthesis temperature 110 °C	4	0.17	6	9.8 × 10^−3^	165 *
AFX-1	[54]	Based on sample Cu-SSZ-16; NaOH conc. in gel x2	4.5	0.21	4.1	6.4 × 10^−3^	263 ± 10
AFX-2	[55]		5.3	0.19	2.1	4.1 × 10^−3^	283 ± 10
AEI-1	[51]		6.2	0.2	2.9	6.1 × 10^−3^	303 ± 10
ERI-CHA-31% CHA	[56]		6.2	0.18	2.4	5.6 × 10^−3^	343 ± 10
ERI-CHA-2 51% CHA	[56]		6.7	0.26	3.3	5.8 × 10^−3^	303 ± 10
ERI-OFF-1	Supplementary Section S3		2.6	0.11	1.9	4.4 × 10^−3^	303 ± 10
BEA-1L	Zeolyst	Cu loading as described in ref. [51]	12.5	0.25	1.4	4.6 × 10^−3^	365 *
BEA-1H	Zeolyst	Cu loading as described in ref. [22]	12.5	0.33	1.3	3.3 × 10^−3^	369 *

* Theoretically calculated *T*_onset_ based on a linear regression.

## References

[1] Locci C., Vervisch L., Farcy B., Domingo P., Perret N. (2018). Selective Non-Catalytic Reduction (SNCR) of Nitrogen Oxide Emissions: A Perspective from Numerical Modeling. Flow Turbul. Combust..

[2] Han L., Cai S., Gao M., Hasegawa J.-Y., Wang P., Zhang J., Shi L., Zhang D. (2019). Selective Catalytic Reduction of NOx with NH3 by Using Novel Catalysts: State of the Art and Future Prospects. Chem. Rev..

[3] Hoffmann A., De Prins M., Sree S.P., Vanbutsele G., Smet S., Chandran C.V., Radhakrishnan S., Breynaert E., Martens J.A. (2022). Selective Catalytic Reduction of NOx with Ammonia (NH3-SCR) over Copper Loaded LEV Type Zeolites Synthesized with Different Templates. Phys. Chem. Chem. Phys..

[4] Borfecchia E., Beato P., Svelle S., Olsbye U., Lamberti C., Bordiga S. (2018). Cu-CHA—A Model System for Applied Selective Redox Catalysis. Chem. Soc. Rev..

[5] Paolucci C., Di Iorio J.R., Schneider W.F., Gounder R. (2020). Solvation and Mobilization of Copper Active Sites in Zeolites by Ammonia: Consequences for the Catalytic Reduction of Nitrogen Oxides. Acc. Chem. Res..

[6] Borfecchia E., Lomachenko K.A., Giordanino F., Falsig H., Beato P., Soldatov A.V., Bordiga S., Lamberti C. (2015). Revisiting the Nature of Cu Sites in the Activated Cu-SSZ-13 Catalyst for SCR Reaction. Chem. Sci..

[7] Shi Z., Peng Q., Jiaqiang E., Xie B., Wei J., Yin R., Fu G. (2023). Mechanism, Performance and Modification Methods for NH3-SCR Catalysts: A Review. Fuel.

[8] Zhang S., Chen J., Meng Y., Pang L., Guo Y., Luo Z., Fang Y., Dong Y., Cai W., Li T. (2022). Insight into Solid-State Ion-Exchanged Cu-Based Zeolite (SSZ-13, SAPO-18, and SAPO-34) Catalysts for the NH3-SCR Reaction: The Promoting Role of NH4-Form Zeolite Substrates. Appl. Surf. Sci..

[9] Khivantsev K., Kwak J.-H., Jaegers N.R., Koleva I.Z., Vayssilov G.N., Derewinski M.A., Wang Y., Aleksandrov H.A., Szanyi J. (2022). Identification of the Mechanism of NO Reduction with Ammonia (SCR) on Zeolite Catalysts. Chem. Sci..

[10] Chen L., Janssens T.V.W., Vennestrøm P.N.R., Jansson J., Skoglundh M., Grönbeck H. (2020). A Complete Multisite Reaction Mechanism for Low-Temperature NH3-SCR over Cu-CHA. ACS Catal..

[11] Bendrich M., Scheuer A., Hayes R.E., Votsmeier M. (2018). Unified Mechanistic Model for Standard SCR, Fast SCR, and NO2 SCR over a Copper Chabazite Catalyst. Appl. Catal. B Environ..

[12] Grossale A., Nova I., Tronconi E., Chatterjee D., Weibel M. (2008). The Chemistry of the NO/NO2–NH3 “Fast” SCR Reaction over Fe-ZSM5 Investigated by Transient Reaction Analysis. J. Catal..

[13] Negri C., Selleri T., Borfecchia E., Martini A., Lomachenko K.A., Janssens T.V.W., Cutini M., Bordiga S., Berlier G. (2020). Structure and Reactivity of Oxygen-Bridged Diamino Dicopper(II) Complexes in Cu-Ion-Exchanged Chabazite Catalyst for NH3-Mediated Selective Catalytic Reduction. J. Am. Chem. Soc..

[14] Gao F., Mei D., Wang Y., Szanyi J., Peden C.H.F. (2017). Selective Catalytic Reduction over Cu/SSZ-13: Linking Homo- and Heterogeneous Catalysis. J. Am. Chem. Soc..

[15] Paolucci C., Parekh A.A., Khurana I., Di Iorio J.R., Li H., Albarracin Caballero J.D., Shih A.J., Anggara T., Delgass W.N., Miller J.T. (2016). Catalysis in a Cage: Condition-Dependent Speciation and Dynamics of Exchanged Cu Cations in Ssz-13 Zeolites. J. Am. Chem. Soc..

[16] Krishna S.H., Goswami A., Wang Y., Jones C.B., Dean D.P., Miller J.T., Schneider W.F., Gounder R. (2023). Influence of Framework Al Density in Chabazite Zeolites on Copper Ion Mobility and Reactivity during NOx Selective Catalytic Reduction with NH3. Nat. Catal. 2023 63.

[17] Paolucci C., Khurana I., Parekh A.A., Li S., Shih A.J., Li H., Di Iorio J.R., Albarracin-Caballero J.D., Yezerets A., Miller J.T. (2017). Dynamic Multinuclear Sites Formed by Mobilized Copper Ions in NOx Selective Catalytic Reduction. Science.

[18] Oda A., Shionoya H., Hotta Y., Takewaki T., Sawabe K., Satsuma A. (2020). Spectroscopic Evidence of Efficient Generation of Dicopper Intermediate in Selective Catalytic Reduction of NO over Cu-Ion-Exchanged Zeolites. ACS Catal..

[19] Liu C., Kubota H., Amada T., Kon K., Toyao T., Maeno Z., Ueda K., Ohyama J., Satsuma A., Tanigawa T. (2020). *In Situ* Spectroscopic Studies on the Redox Cycle of NH3−SCR over Cu−CHA Zeolites. ChemCatChem.

[20] Hu W., Selleri T., Gramigni F., Fenes E., Rout K.R., Liu S., Nova I., Chen D., Gao X., Tronconi E. (2021). On the Redox Mechanism of Low-Temperature NH3-SCR over Cu-CHA: A Combined Experimental and Theoretical Study of the Reduction Half Cycle. Angew. Chemie Int. Ed..

[21] Nielsen D., Gao Q., Janssens T.V.W., Vennestrøm P.N.R., Mossin S. (2023). Cu-Speciation in Dehydrated CHA Zeolites Studied by H 2 -TPR and *In Situ* EPR. J. Phys. Chem. C.

[22] Wu Y., Zhao W., Ahn S.H., Wang Y., Walter E.D., Chen Y., Derewinski M.A., Washton N.M., Rappé K.G., Wang Y. (2023). Interplay between Copper Redox and Transfer and Support Acidity and Topology in Low Temperature NH3-SCR. Nat. Commun..

[23] Göltl F., Sautet P., Hermans I. (2016). The Impact of Finite Temperature on the Coordination of Cu Cations in the Zeolite SSZ-13. Catal. Today.

[24] Göltl F., Sautet P., Hermans I. (2015). Can Dynamics Be Responsible for the Complex Multipeak Infrared Spectra of NO Adsorbed to Copper(II) Sites in Zeolites?. Angew. Chem. Int. Ed..

[25] Zhang R., McEwen J.S., Kollár M., Gao F., Wang Y., Szanyi J., Peden C.H.F. (2014). NO Chemisorption on Cu/SSZ-13: A Comparative Study from Infrared Spectroscopy and DFT Calculations. ACS Catal..

[26] Lee H., Song I., Jeon S.W., Kim D.H. (2021). Mobility of Cu Ions in Cu-SSZ-13 Determines the Reactivity of Selective Catalytic Reduction of NOxwith NH3. J. Phys. Chem. Lett..

[27] Godiksen A., Vennestrøm P.N.R., Rasmussen S.B., Mossin S. (2017). Identification and Quantification of Copper Sites in Zeolites by Electron Paramagnetic Resonance Spectroscopy. Top. Catal..

[28] Lei H., Rizzotto V., Guo A., Ye D., Simon U., Chen P. (2021). Recent Understanding of Low-Temperature Copper Dynamics in Cu-Chabazite NH3-SCR Catalysts. Catalysts.

[29] Iacobone U., Nova I., Tronconi E., Villamaina R., Ruggeri M.P., Collier J., Thompsett D. (2022). Appraising Multinuclear Cu^2+^ Structure Formation in Cu-CHA SCR Catalysts via Low-T Dry CO Oxidation with Modulated NH3 Solvation. ChemistryOpen.

[30] Chen P., Khetan A., Jabłońska M., Simböck J., Muhler M., Palkovits R., Pitsch H., Simon U. (2018). Local Dynamics of Copper Active Sites in Zeolite Catalysts for Selective Catalytic Reduction of NOx with NH3. Appl. Catal. B Environ..

[31] Chen P., Rizzotto V., Khetan A., Xie K., Moos R., Pitsch H., Ye D., Simon U. (2019). Mechanistic Understanding of Cu-CHA Catalyst as Sensor for Direct NH 3 -SCR Monitoring: The Role of Cu Mobility. ACS Appl. Mater. Interfaces.

[32] Kwak J.H., Varga T., Peden C.H.F., Gao F., Hanson J.C., Szanyi J. (2014). Following the Movement of Cu Ions in a SSZ-13 Zeolite during Dehydration, Reduction and Adsorption: A Combined *in situ* TP-XRD, XANES/DRIFTS Study. J. Catal..

[33] Millan R., Cnudde P., Hoffman A.E.J., Lopes C.W., Concepcion P., Van Speybroeck V., Boronat M. (2020). Theoretical and Spectroscopic Evidence of the Dynamic Nature of Copper Active Sites in Cu-CHA Catalysts under Selective Catalytic Reduction (NH3-SCR-NOx) Conditions. J. Phys. Chem. Lett..

[34] Rizzotto V., Chen P., Simon U. (2018). Mobility of NH3-Solvated CUII Ions in Cu-SSZ-13 and Cu-ZSM-5 NH3-SCR Catalysts: A Comparative Impedance Spectroscopy Study. Catalysts.

[35] Millan R., Cnudde P., van Speybroeck V., Boronat M. (2021). Mobility and Reactivity of Cu + Species in Cu-CHA Catalysts under NH 3 -SCR-NOx Reaction Conditions: Insights from AIMD Simulations. JACS Au.

[36] Kocman V., Di Mauro G.M., Veglia G., Ramamoorthy A. (2019). Use of Paramagnetic Systems to Speed-up NMR Data Acquisition and for Structural and Dynamic Studies. Solid State Nucl. Magn. Reson..

[37] Pell A.J., Pintacuda G., Grey C.P. (2019). Progress in Nuclear Magnetic Resonance Spectroscopy Paramagnetic NMR in Solution and the Solid State. Prog. Nucl. Magn. Reson. Spectrosc..

[38] Li W., Zhang Q., Joos J.J., Smet P.F., der Günne J.S.A. (2019). Blind Spheres of Paramagnetic Dopants in Solid State NMR. Phys. Chem. Chem. Phys..

[39] Parigi G., Benda L., Ravera E., Romanelli M., Luchinat C. (2019). Pseudocontact Shifts and Paramagnetic Susceptibility in Semiempirical and Quantum Chemistry Theories. J. Chem. Phys..

[40] Boddenberg B., Hartmann M. (1995). Proton Magnetic Resonance of Cu(II) Ammonia Complexes in Zeolite Y. Z. Fur Phys. Chem..

[41] Grey C.P., Vega A.J. (1995). Determination of the Quadrupole Coupling Constant of the Invisible Aluminum Spins in Zeolite HY with 1H/27A1 TRAPDOR NMR. J. Am. Chem. Soc..

[42] Vallaey B., Radhakrishnan S., Heylen S., Chandran C.V., Taulelle F., Breynaert E., Martens J.A. (2018). Reversible Room Temperature Ammonia Gas Absorption in Pore Water of Microporous Silica-Alumina for Sensing Applications. Phys. Chem. Chem. Phys..

[43] Hunger M. (1997). Brønsted Acid Sites in Zeolites Characterized by Multinuclear Solid-State NMR Spectroscopy. Catal. Rev. Sci. Eng..

[44] Haase F., Sauer J. (1994). 1H NMR Chemical Shifts of Ammonia, Methanol, and Water Molecules Interacting with Brønsted Acid Sites of Zeolite Catalysts: Ab-Initio Calculations. J. Phys. Chem..

[45] De Prins M., Verheyen E., Hoffmann A., Vanbutsele G., Sree S.P., Kerkhofs S., Van Tendeloo L., Schütze F.W., Martens J. (2020). Structural Parameters Governing Low Temperature Activity of Small Pore Copper Zeolites in NH3-SCR. J. Catal..

[46] De Prins M., Verheyen E., Kerkhofs S., Hoffmann A., Vanbutsele G., Sree S.P., Radhakrishnan S., Van Tendeloo L., Breynaert E., Kirschhock C.E.A. (2018). EU-7 Zeolite: A Synthetic BIK Type Zeolite with High Hydrothermal Stability. Chem. Commun..

[47] Hahn E.L. (1950). Spin Echoes. Phys. Rev..

[48] Feike M., Demco D.E., Graf R., Gottwald J., Hafner S., Spiess H.W. (1996). Broadband Multiple-Quantum NMR Spectroscopy. J. Magn. Reson. Ser. A.

[49] Jeener J., Meier B.H., Bachmann P., Ernst R.R. (1979). Investigation of Exchange Processes by Two-dimensional NMR Spectroscopy. J. Chem. Phys..

[50] Bräuniger T., Wormald P., Hodgkinson P. (2002). Improved Proton Decoupling in NMR Spectroscopy of Crystalline Solids Using the SPINAL-64 Sequence. Monatshefte Fur Chem..

[51] Hoffmann A., De Prins M., Smet S., Sree S.P., Verheyen E., Martens J., Van Tendeloo L., Schütze F.-W. (2019). Stable Small-Pore Zeolites.

[52] Verheyen E., Sree S.P., Smet S., Hoffmann A., De Prins M., Martens J., Van Tendeloo L., Schütze F.-W. (2017). Stable CHA Zeolites.

[53] Martens J., Sree S.P., Kerkhofs S., Verheyen E., Schütze F.-W. (2019). One-Pot Synthesis of Copper Containing Small-Pore Zeolites.

[54] Fickel D.W., Lobo R.F. (2010). Copper Coordination in Cu-SSZ-13 and Cu-SSZ-16 Investigated by Variable-Temperature XRD. J. Phys. Chem. B.

[55] Hrabanek P., Zikanova A., Supinkova T., Drahokoupil J., Fila V., Lhotka M., Dragounova H., Laufek F., Brabec L., Jirka I. (2016). Static In-Situ Hydrothermal Synthesis of Small Pore Zeolite SSZ-16 (AFX) Using Heated and Pre-Aged Synthesis Mixtures. Microporous Mesoporous Mater..

[56] Sree S.P., Verheyen E., De Prins M., Van Der Donck T., Van Tendeloo L., Schuetze F., Martens J.A. (2021). Synthesis of a New Zeolite, Intergrowth of Erionite and Chabazite. ACS Mater. Lett..

