# Supplementary material for: Prediction of Cu Zeolite NH3-SCR Activity from Variable Temperature 1H NMR Spectroscopy
Source: Molecules. 2023 Sep 6;28(18):6456. doi: 10.3390/molecules28186456 (PMC10537069; doi:10.3390/molecules28186456)
Supplement: Supplementary file 1 [file molecules-28-06456-s001.zip › molecules-2557072-supplementary.pdf]

Supplementary Materials

S1. Extended NMR data discussed in the manuscript

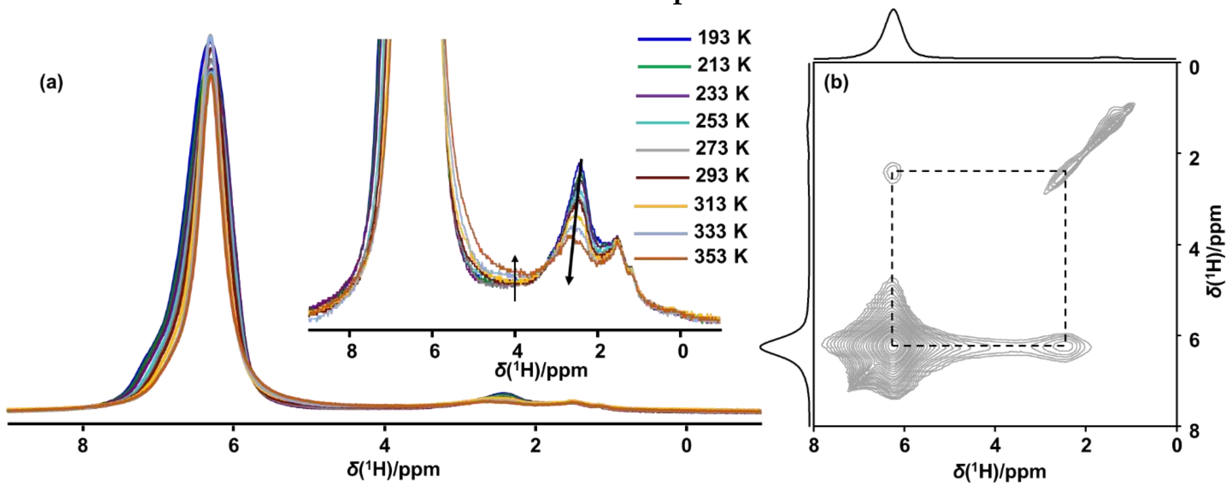

Figure S1. (a) Variable temperature ^{1}H NMR spectrum of NH_4-exchanged CHA-1 zeolite. A zoomed in picture is provided in the inset to show the changes in individual resonances with increasing temperature. (b) ^{1}H-^{1}H EXSY spectrum of NH_4-exchanged CHA-1 zeolite, showing the existence of chemical exchange between aluminol species at 2.5 ppm and NH_4^+ resonance at 6.5 ppm.

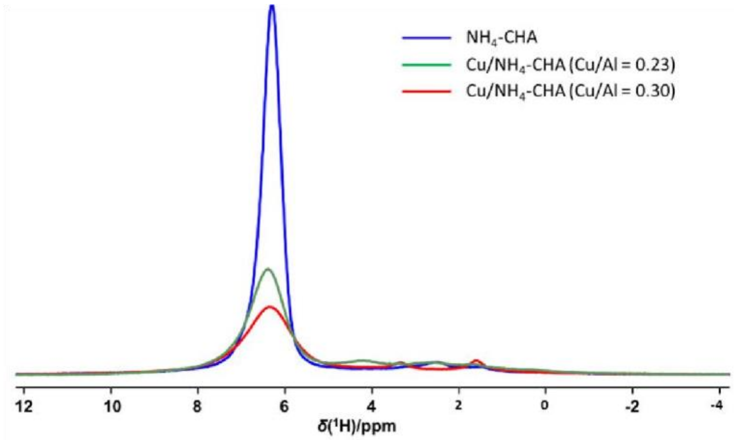

Figure S2. ^{1}H NMR spectrum of a CHA-2 zeolite in its NH_4-form (blue) and Cu-exchanged forms with Cu/Al ratios of 0.23 (green) and 0.30 (red) at 293 K.

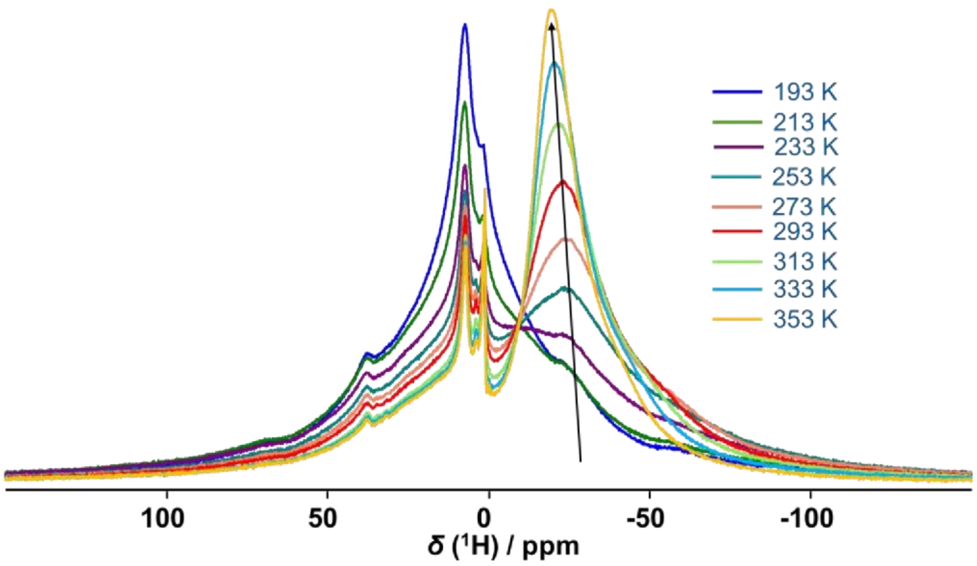

Figure S3. Variable temperature ^{1}H NMR spectra of NH_3 exposed Cu-CHA-1H catalyst. The resonance attributed to NH_3 adsorbed on Cu^{II} ions that underwent paramagnetic shift to negative chemical shifts, show the influence of temperature on the paramagnetic shift.

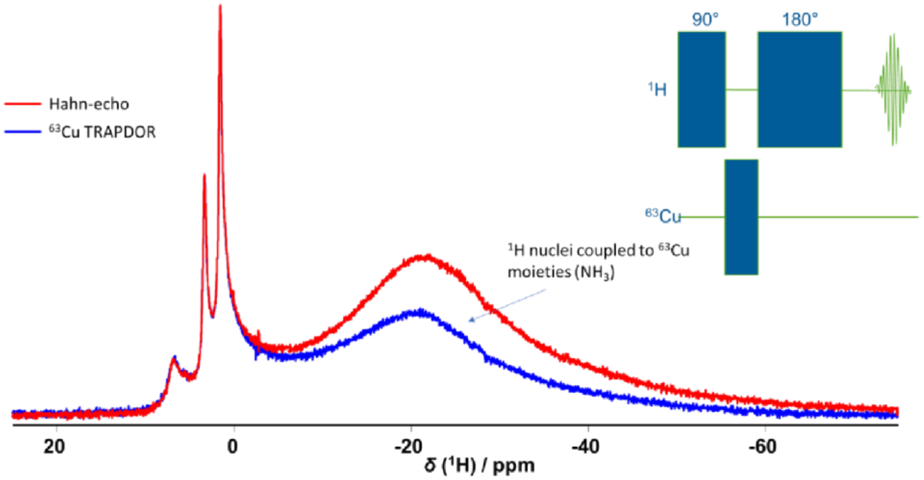

Figure S4. Comparison of ^{1}H Hahn-echo and $^{1}H/^{63}Cu$ TRAPDOR NMR spectra of NH_3-exposed CHA-2H catalyst. A schematic of the NMR pulse sequence is provided in the inset.

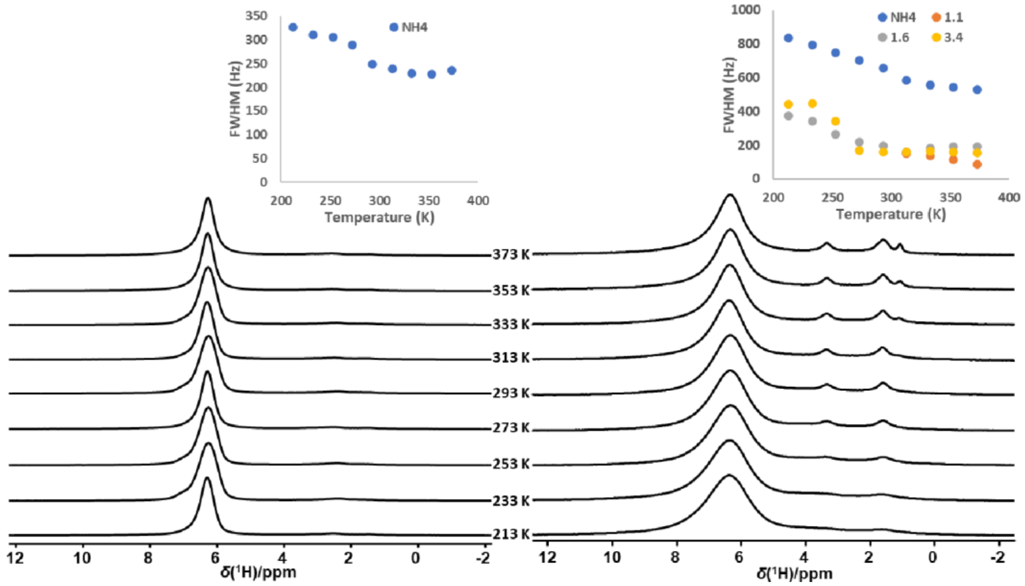

Figure S5. Variable temperature (VT) ^{1}H NMR spectra of (a) NH_4-exchanged CHA-2, *Inset:* FWHM of the NH_4resonance as function of temperature and; (b) Cu-exchanged CHA-2 (CHA-2H Cu/Al 0.3). *Inset:* FWHM of the NH_4resonance and suddenly appearing resonances at ~1.1 ppm, 1.6 ppm and 3.4 ppm respectively)

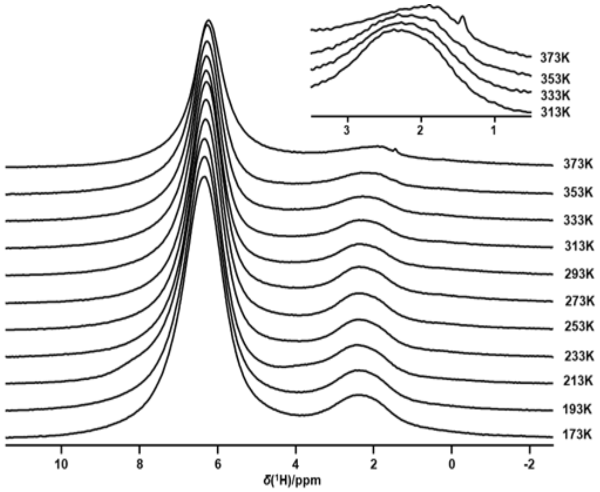

Figure S6. Variable temperature ^{1}H NMR spectra of Cu/NH_4-CHA-1L. A magnification of the 0.5 – 3.5 ppm for temperatures between 313 K – 373 K is shown in the inset to show the transition.

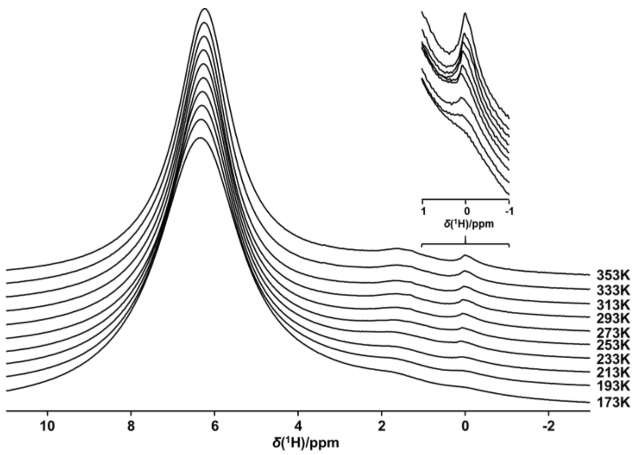

Figure S7. Variable temperature ^{1}H NMR spectra of Cu/NH_4-CHA-8. The resonance at ca. 0 ppm that undergoes sharpening with increase in temperature is visible as a shoulder even at 173 K, the limit of the NMR probehead.

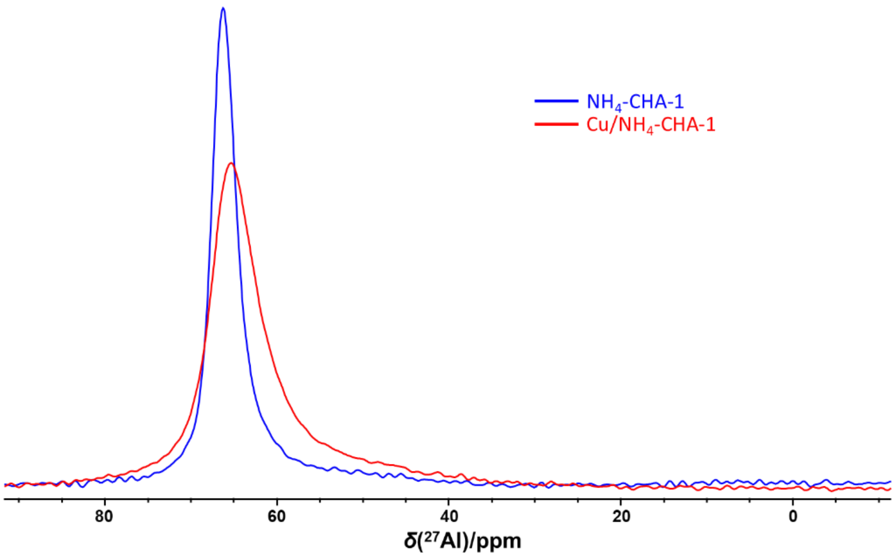

Figure S8. ^{1}H decoupled ^{27}Al NMR spectrum of CHA-1 in NH_4-form (blue trace) and partially Cu-exchanged form (red trace) recorded at 295 K.

S2. Characterization of the different zeolite catalysts

CHA-1

Micropore volume: 0.216 cm^3/g

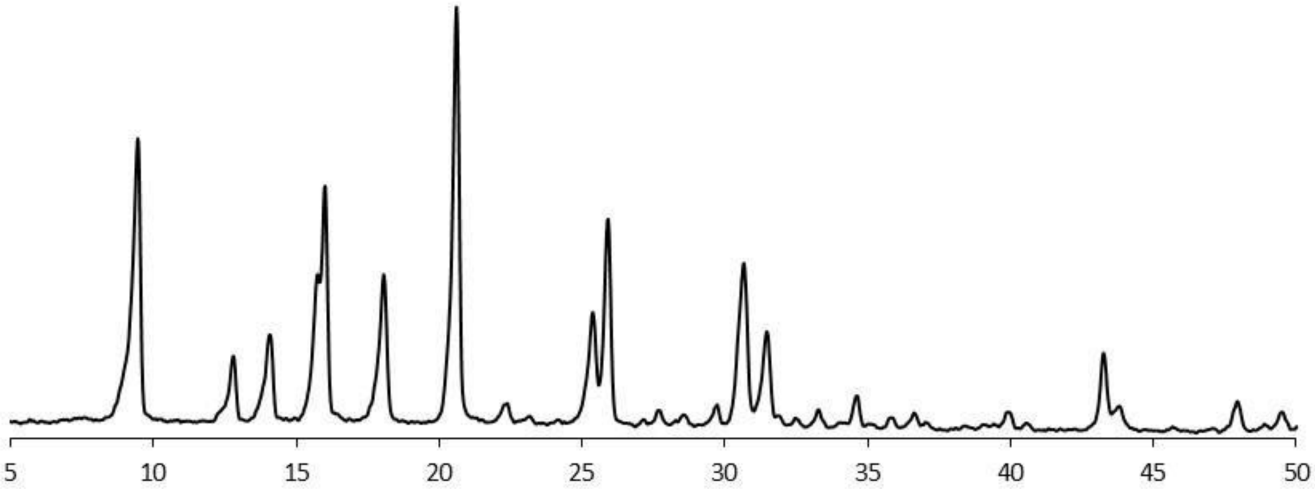

Figure S9. X-Ray Diffractogram of CHA-1

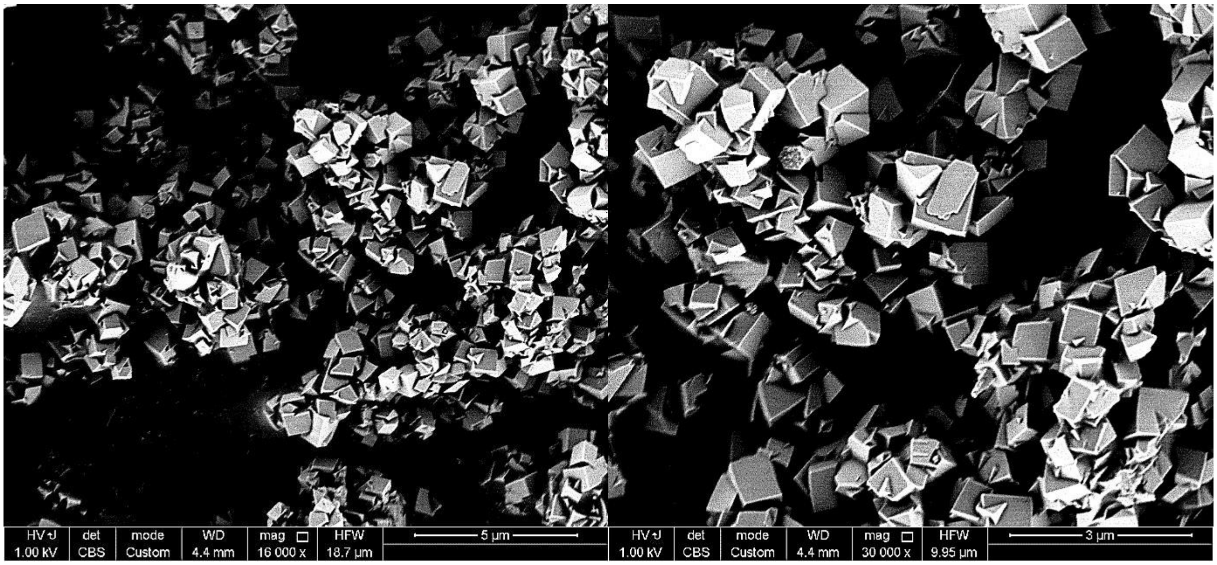

Figure S10. SEM images of CHA-1

CHA-2

Micropore volume: 0.246 cm^3/g

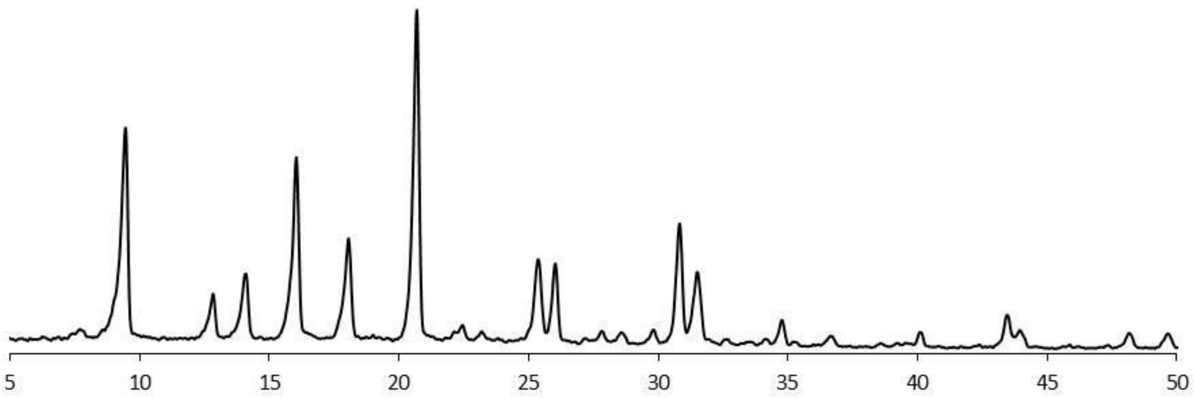

Figure S11. X-Ray Diffractogram of CHA-2

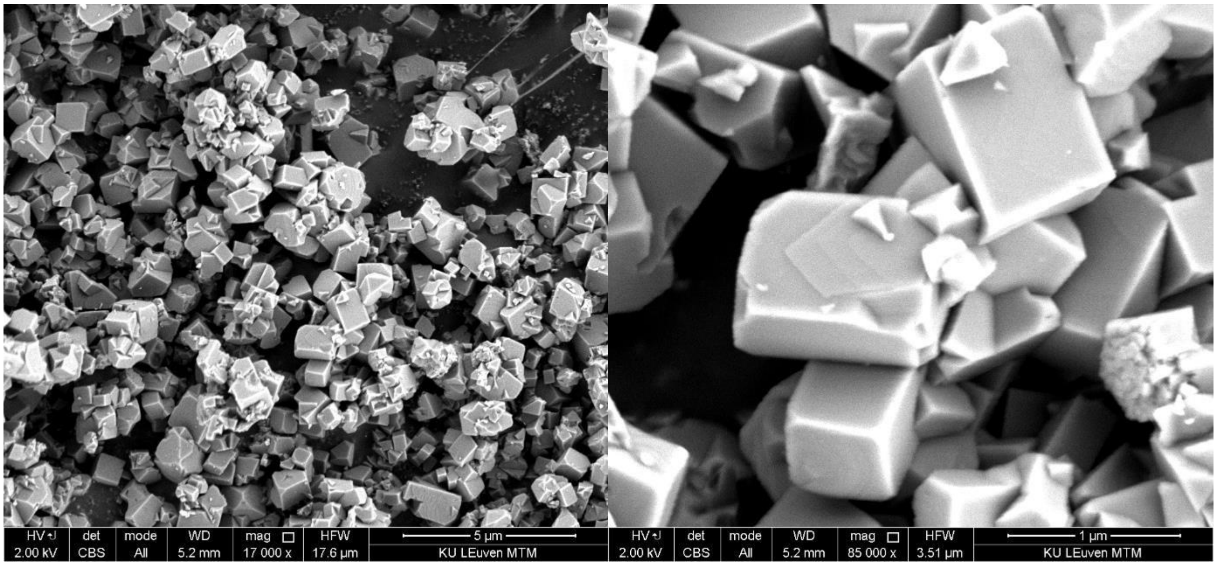

Figure S12. SEM images of CHA-2

CHA-3

Micropore volume : 0.228 cm^3/g

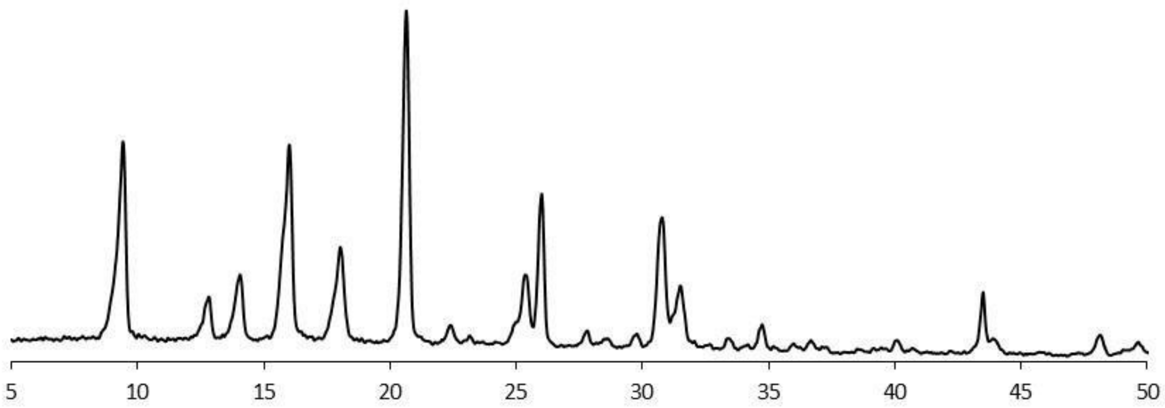

Figure S13. X-Ray Diffractogram of CHA-3

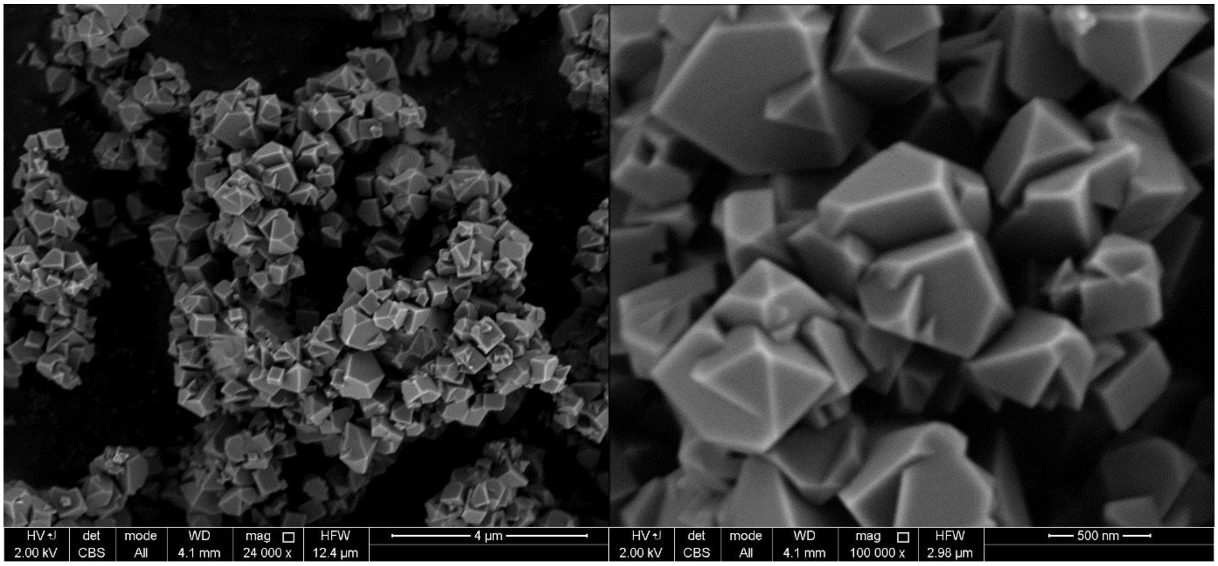

Figure S14. SEM images of CHA-3

CHA-4 (GV440)

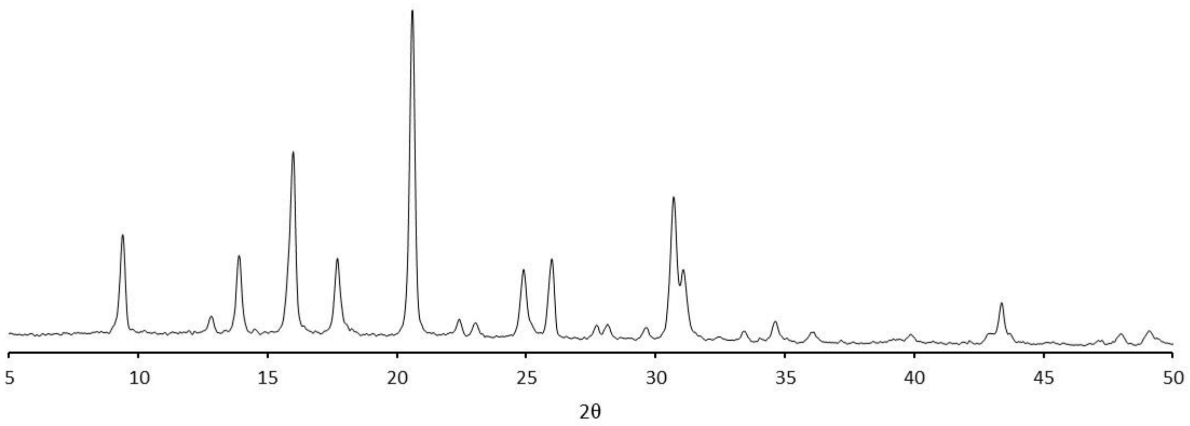

Figure S15. X-Ray Diffractogram of CHA-4

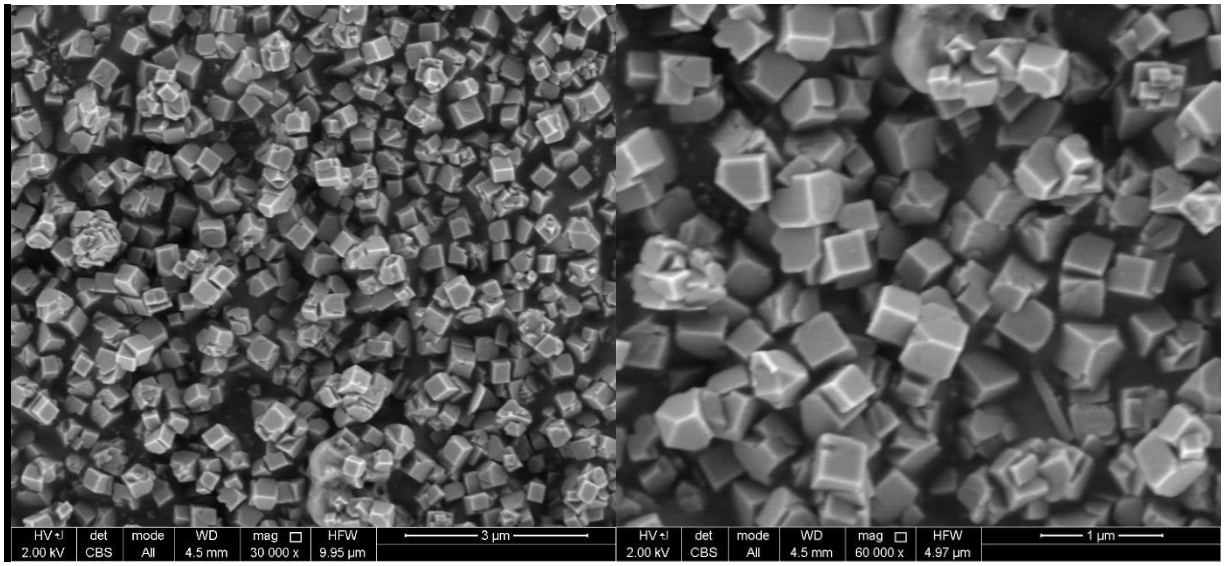

Figure S16. SEM images of CHA-4

CHA-5

Micropore volume : 0.242 cm^3/g

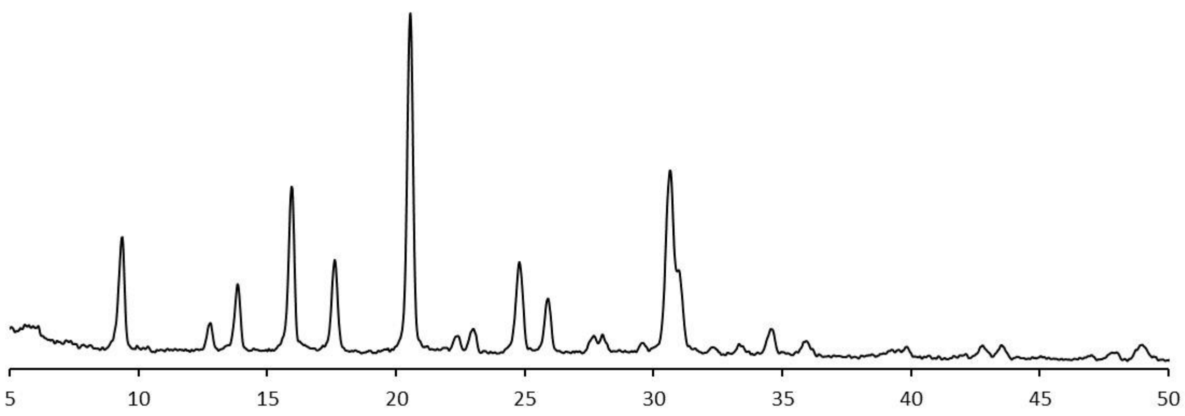

Figure S17. X-Ray Diffractogram of CHA-5

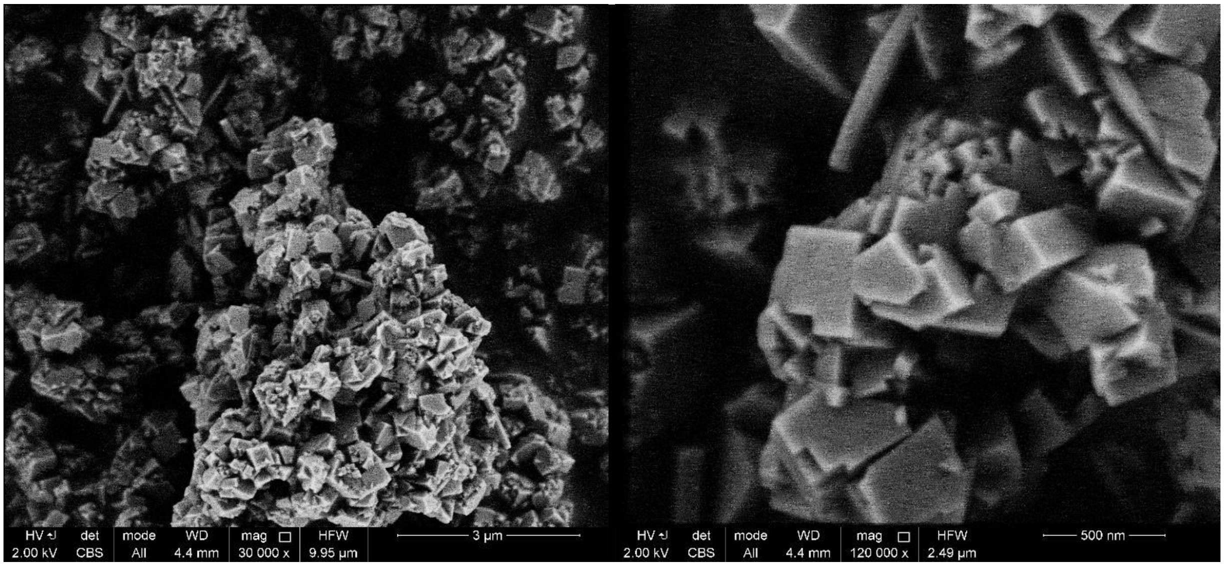

Figure S18. SEM images of CHA-5

CHA-6

Micropore volume : 0.210 cm^3/g

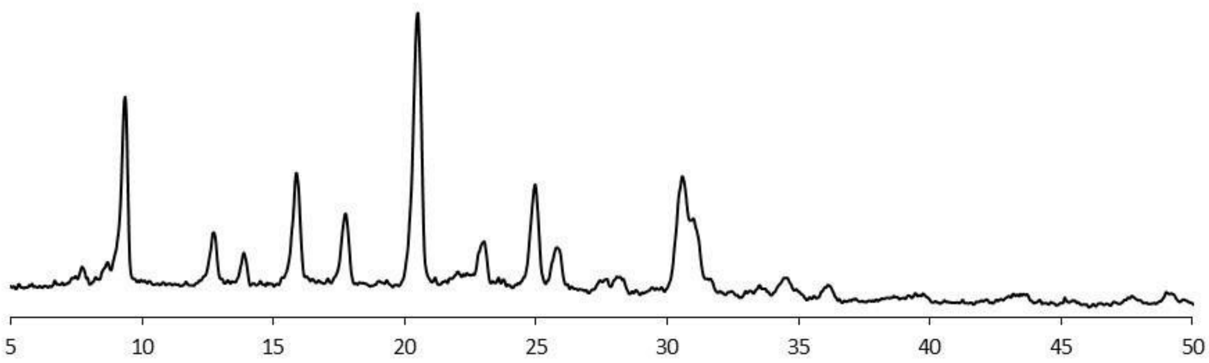

Figure S19. X-Ray Diffractogram of CHA-6

CHA-8

Micropore volume : 0.200 cm^3/g

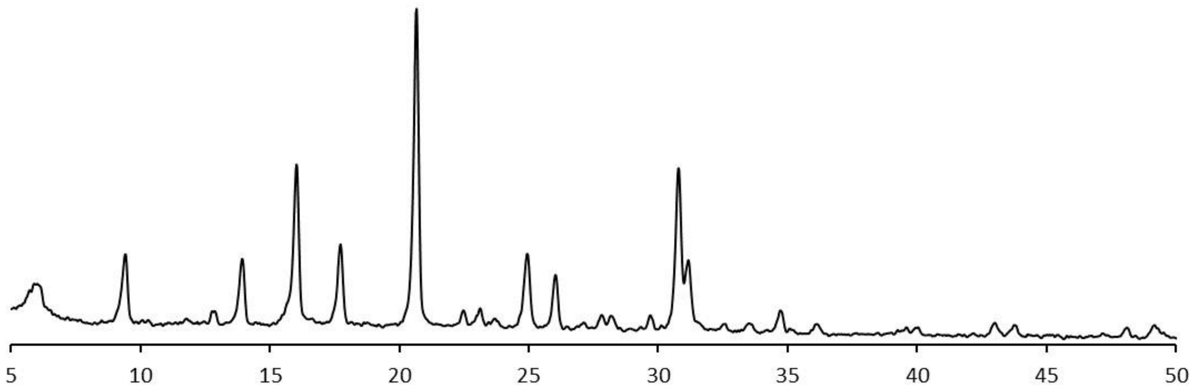

Figure S20. X-Ray Diffractogram of CHA-8

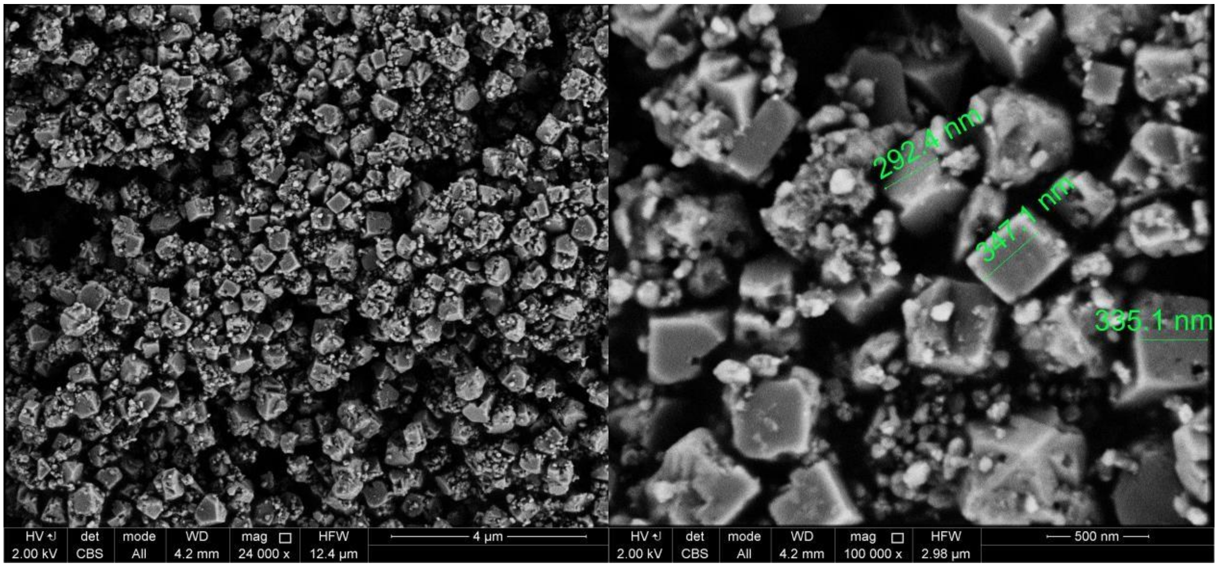

Figure S21. SEM images of CHA-8

AFX-1

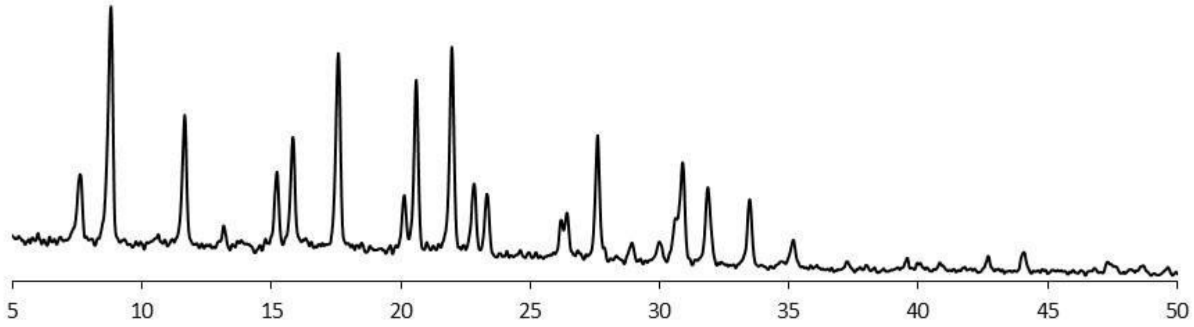

Figure S22. X-Ray Diffractogram of AFX-1

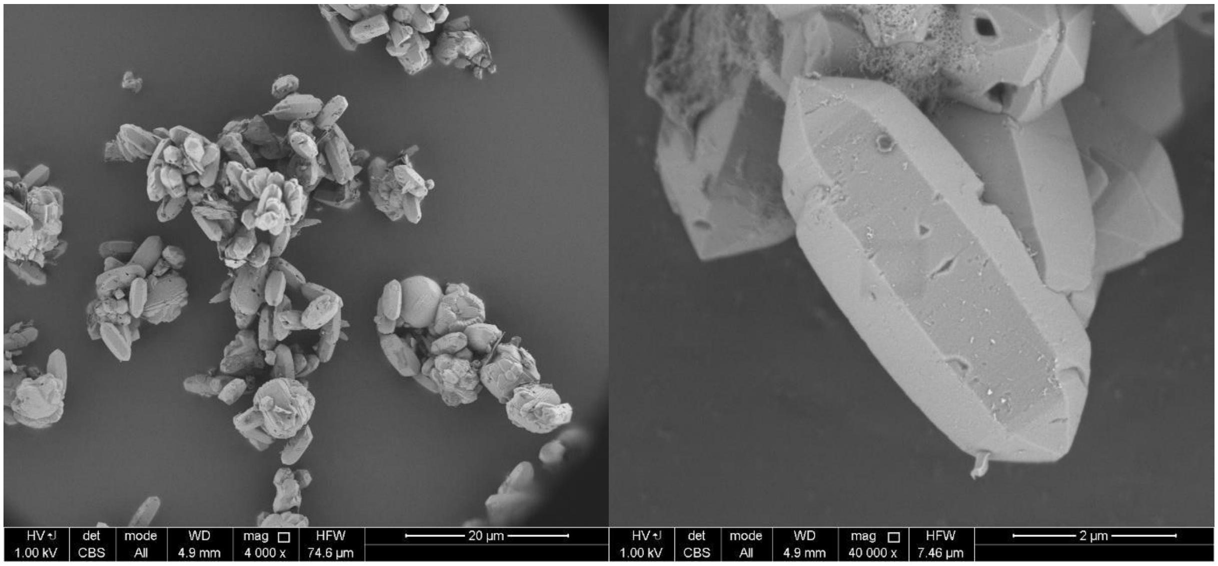

Figure S23. SEM images of AFX-1

AFX-2

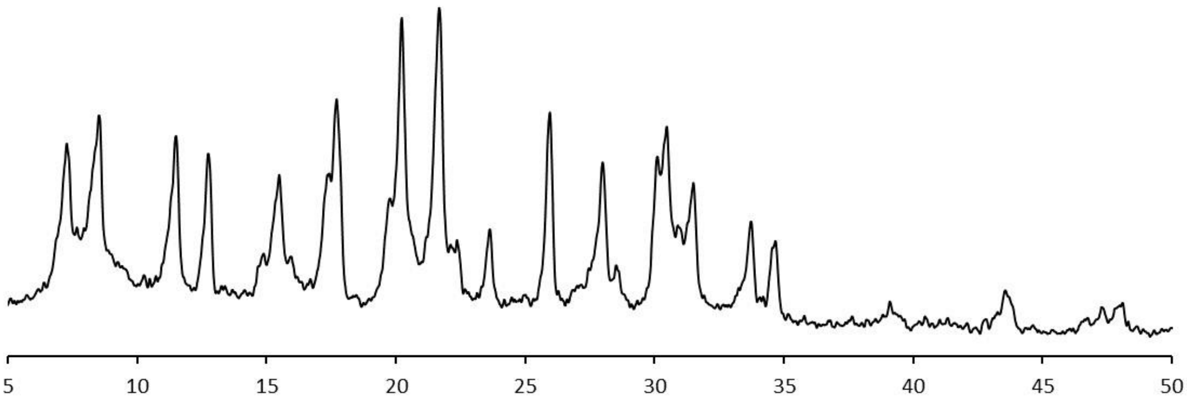

Figure S24. X-Ray Diffractogram of AFX-2 *AEI-1*

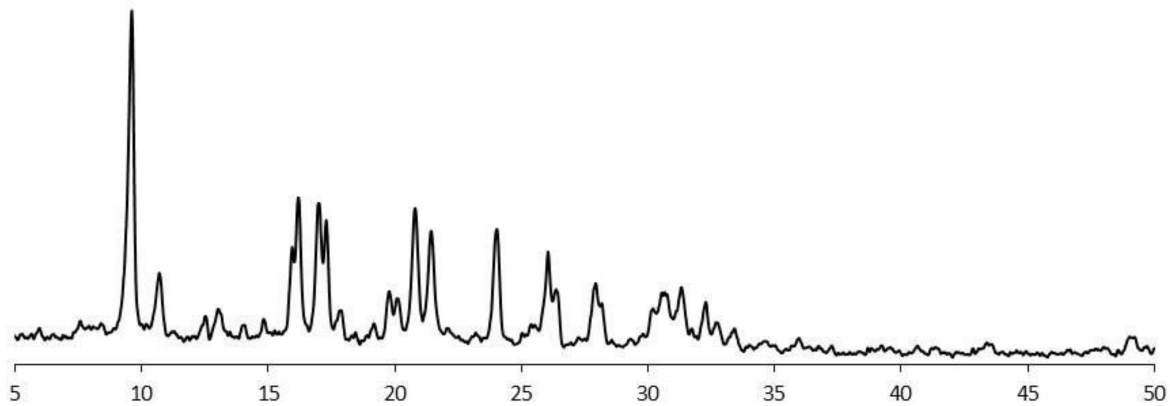

Figure S25. X-Ray Diffractogram of AEI-1

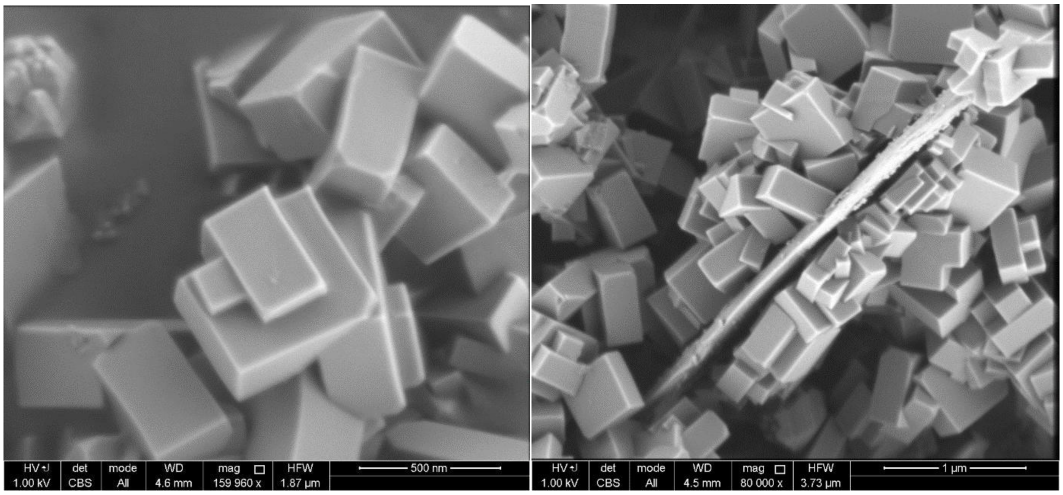

Figure S26. SEM images of AEI-1

ERI-CHA-1

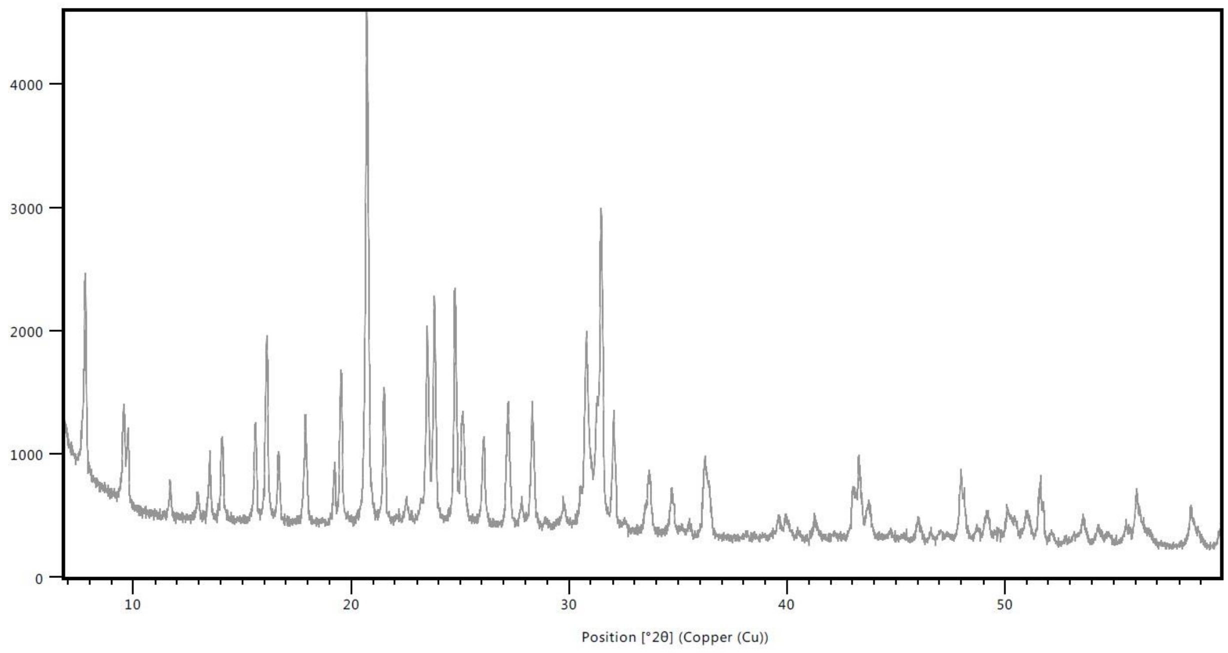

Figure S27. X-Ray Diffractogram of ERI-CHA-1 *ERI-CHA-2*

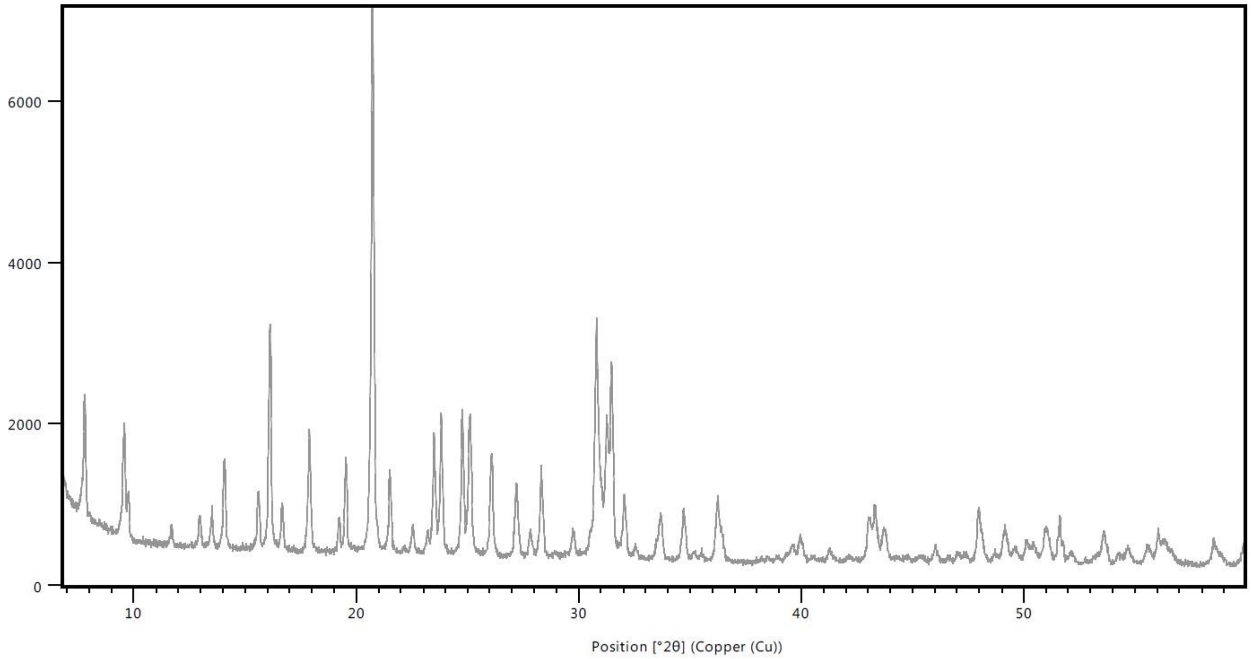

Figure S28. X-Ray Diffractogram of ERI-CHA-2

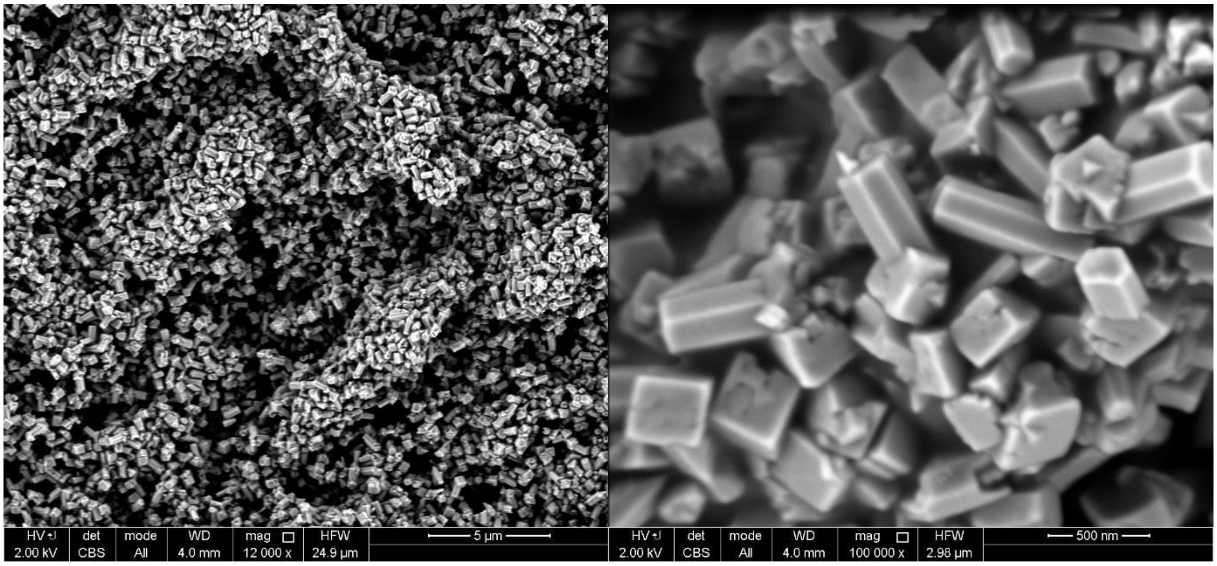

Figure S29. SEM images of ERI-CHA-2

ERI-OFF-1

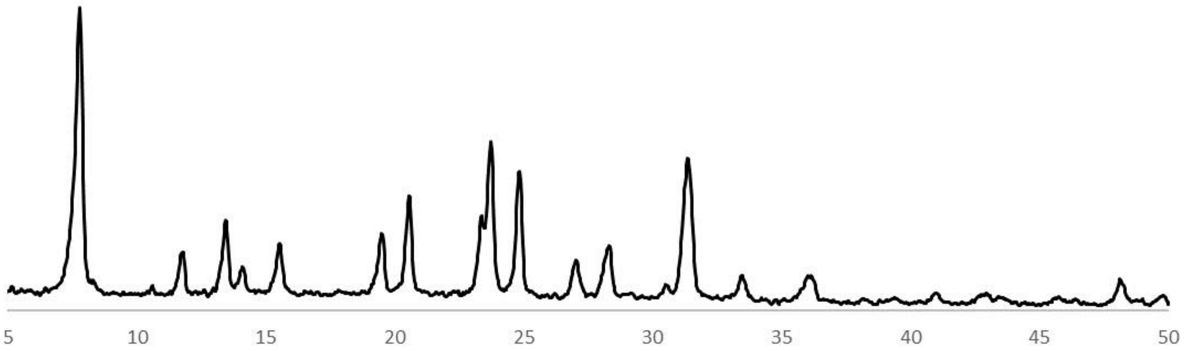

Figure S30. X-Ray Diffractogram of ERI-OFF-1

<u>S3. Synthesis of ERI-OFF-1</u>

A solution was made by adding potassium hydroxide (Fisher Scientific), aluminium hydroxide ($Al(OH)_3$; BDH Chemicals), strontium nitrate ($Sr(NO_3)_2$ – Acros), Ludox HS-40 (Sigma-Aldrich) and milliQ water. This mixture was stirred for 60 h at room temperature.

A second solution was made by adding TEAOH (35 wt% - Sigma-Aldrich), Al-sec-butoxide (Fluka), Ludox AS-40 (Sigma-Aldrich) and milliQ water. This mixture was stirred and then heated to 95 °C for 18 h in static conditions. TMACl (Sigma-Aldrich) was added and the mixture was stirred for 60 h at room temperature.

The two mixtures were homogenized for 10 min and transferred to a stainless steel autoclave. The final gel has the following molar ratios : SiO_2 / 0.082 Al_2O_3 / 0.33 KOH / 0.32 TEAOH / 0.0060 Sr / 0.021 TMA / 16 H_2O. This mixture was heated for 168 h at 180 °C under static conditions. The solid product was recovered by filtration and washing, and was dried at 60 °C for 16 h. The sample was then calcined under nitrogen flow at 550 °C for 5 h (heating rate: 5 °C/min). Upon cooling down to 200 °C, the gas flow was switched to oxygen and the sample was heated again to 550 °C for 16 h (heating rate: 5 °C/min). After calcination the sample was suspended in a 0.5M ammonium chloride solution (MP Biomedicals LLC) (100 ml solution / 1 g of sample) and mixed for 4 h under reflux conditions. The material was recovered by filtration and dried at 60 °C for 16 h. This exchange procedure was repeated an additional two times. Finally the sample was suspended in an aqueous solution of copper (II) acetate (Sigma-Aldrich) (0.01885 g of copper (II) acetate in 200 ml of milliQ water) (100 ml of solution / 1 g of sample) and stirred for 24 h at room temperature. The sample was recovered by filtration and dried at 60 °C for 16 h.
